# Establishing molecular biomarkers for efficient demarcation of tumor and non-tumor tissue in oral squamous cell carcinoma

**DOI:** 10.1038/s41598-025-33758-1

**Published:** 2025-12-26

**Authors:** Kashish Gupta, Ishan Raval, Apurvasinh Puvar, Shayma Shaikh, Madhvi Joshi, Chaitanya Joshi, Siddharth Shah, Anand Shah, Shashank Pandya

**Affiliations:** 1 Gujarat Biotechnology Research Centre (GBRC), Gandhinagar, 382011 Gujarat India; 2 Zydus Cancer Hospital, Ahmedabad, 380059 India; 3 The Gujarat Cancer and Research Institute (GCRI), Ahmedabad, 380016 India

**Keywords:** OSCC (Oral squamous cell carcinoma), HNSC (Head and neck squamous cell carcinoma), Differentially expressed genes (DEG), Biomarker, Margin clearance, Biomarkers, Cancer, Computational biology and bioinformatics, Oncology

## Abstract

Oral squamous cell carcinoma (OSCC), which accounts for 90% of head and neck cancers **(**HNSC), is a prevalent cancer, especially in India, where it ranks among the top three cancers. Despite treatment advancements, OSCC incidence is rising, and recurrence remains a major concern. To improve patient prognosis, effective biomarkers for tumor demarcation are crucial. RNA isolation, library preparation, and RNA sequencing were performed on tumor and adjacent normal oral tissue samples. Transcriptomic analysis identified differentially expressed genes (DEGs) between tumor and normal tissues, which may serve as potential biomarkers. These findings were subsequently validated using RT-qPCR. The analysis revealed 704 upregulated and 1540 downregulated genes. Among these from the top 100 upregulated and downregulated genes, 15 and 9 genes respectively were also reported in the HNSC database of TCGA (The Cancer Genome Atlas). To identify potential biomarkers, the study evaluated multiple factors, including log2 fold change, average RPKM, ROC curve analysis, protein-protein interaction (PPI) network analysis and gene ontology (GO) analysis. The differential expression across various cancer stages and individual sample comparisons were also assessed. The upregulated genes *MMP1*, *MMP10*, *MMP13*, *MMP3*, *ADAM12*, *IL24*, and *ISG15* demonstrated potential as biomarkers, with the highest log2 fold change, average RPKM, and AUC values. These significant genes could be valuable biomarkers for efficient demarcation between tumor and non-tumor tissues in oral cancer. This could lead to improved margin clearance, addressing concerns related to high recurrence rate of OSCC and ultimately enhance patient prognosis.

**Supplementary Information:**

The online version contains supplementary material available at 10.1038/s41598-025-33758-1.

## Introduction

 Oral squamous cell carcinoma (OSCC) accounts for over 90% of the head and neck cancers^1^ and 94% of the oral cavity cancers worldwide^2^. It is the 16th most leading cancer in the world amongst both sexes and all ages and ranks 16th and 15th for incidence and mortality rates respectively^3^. The problem is significantly higher in India as it is one of the top 3 leading cancers with being the top and fourth most frequent cancer for males and females respectively. It has an incidence rate of 9.9 and mortality rate of 5.6%^4^. India has one third of the oral cancer cases in the world^5^ and oral cancer accounts for around 30% of all cancers in India^6^.

Oral cancer affects various sites like the tongue, cheeks, palate, lips, and gingiva, significantly impacting swallowing, chewing, breathing, and speech, and posing a life-threatening risk^7^. Tobacco use is a key factor in OSCC occurrence and progression^8^. Despite advancements in treatment and screening, OSCC incidence is rising, with high mortality and morbidity^9–11^. Early detection improves survival, yet most cases are diagnosed at advanced stages, reducing prognosis^12^. Treatments like surgery, chemotherapy, and radiotherapy exist, but the five-year survival rate remains below 50%^13,14^. Locoregional recurrence affects 60% of advanced and 30% of early OSCC cases, with recurrence rates higher in patients with histologic positive tumor margins, indicating the need for better predictive markers^15^.

Margin status is an important prognostic factor for OSCC, and suitable surgical resection is vital for local control and prognosis^16^. During oral cancer surgery, it is important to achieve adequate resection margins to improve patient prognosis. Surgeons have this important task of attaining adequate resection and conserving reasonable remaining function and satisfactory physical appearance, while relying only on preoperative imaging, visual inspection, and palpation^17^. However, the definition of “clear margin” stays debatable^16^. To improve patient’s outcome, the attainment of clear margins should be considered an important surgical goal and therefore a solution should be sought that can rapidly evaluate the entire resection surface^18^. Thus, it is important to identify effective biomarkers which could demarcate tumor and non-tumor tissue for efficient margin clearance. This has potential implications for approaches tailored to the individual level.

In this study we identified differentially expressed genes (upregulated and downregulated) between the tumor and adjacent normal oral tissue samples which are significant in OSCC. These significant genes have the potential to be biomarkers for efficient margin clearance during surgery which can help manage the concerns of high recurrence rate of OSCC.

## Materials and methods

### Collection of clinical samples

Oral squamous cell carcinoma (OSCC) and their adjacent normal tissue samples were collected with patient consent and ethical approval from Zydus Cancer Hospital and Gujarat Cancer and Research Institute (GCRI), Ahmedabad, Gujarat, India in the years 2021–2024. The study was approved by the GCRI/GCS Ethics Committee-BHR numbered EC-BHR-O-10-2024 (dated 12-07-2024) and the Zydus Ethics Committee (dated 9th Jan 2021) working in accordance to ICH-GCP, the New Drugs and Clinical Trial Rules, 2019, ICMR guidelines, and other applicable regulations. The samples were kept in RNAlater and stored at -80 °C. Sample details such as gender, age, tobacco consumption habits and cancer stage were characterized during sample collection (Supplementary Table 1) with consent from all the patients.

### RNA isolation

RNA isolation from all tumor and adjacent normal oral tissue samples was performed using the Qiagen RNeasy Plus Mini Kit, following the manufacturer’s protocol. Quantification of the isolated RNA was conducted by Qubit™ (Qubit™ RNA HS Assay Kit) and QIAxpert™, and its integrity was assessed using the Bioanlyzer™ (Agilent RNA 6000 Nano Kit) and QIAxcel™. Samples with lower RNA concentrations (< 10ng/µL) or compromised integrity were excluded, while the remaining samples proceeded to library preparation.

### Library Preparation and RNA sequencing

After RNA isolation, libraries for RNA Sequencing were prepared using the Illumina TruSeq Stranded Total RNA kit (Illumina, CA, USA), following the manufacturer’s protocol. Ribosomal RNA (rRNA) was removed followed by RNA fragmentation and cDNA synthesis. The 3’ ends were adenylated for adapter ligation, and enriched DNA fragments were purified to create the final cDNA library. Library concentration was measured using Qubit™ (Qubit™ DNA HS Assay Kit), and integrity was assessed with the Bioanalyzer™ (Agilent High Sensitivity DNA Kit) and QIAxcel™. Samples with lower library concentration (< 5ng/µL) or compromised integrity were excluded, while the remaining samples proceeded to next-generation RNA sequencing. Sequencing was performed on the Illumina NovaSeq platform which gave an average of ~ 11 million reads per sample. Processed reads were used for transcriptomic analysis, and the raw data for all oral tumor and adjacent normal samples are available on NCBI under the BioProject PRJNA1127288. Consequently, data for 32 tumor and 27 adjacent normal samples were obtained and further processed for transcriptomic analysis.

### Transcriptomic analysis

After RNA sequencing, transcriptomic analysis began with quality assessment using FastQC. Reads were mapped to the human genome (GRCh38.p13) in CLC Genomics Workbench (version 12.0.3, Qiagen Bioinformatics Licensed to GBRC). A PCA plot was generated to detect and remove outliers. Analysis was conducted in stages, including the full dataset, cancer stage-based evaluation, and individual sample comparisons.

#### Comprehensive transcriptomic analysis of the complete dataset

After conducting mapping and PCA plot analysis, differential expression analysis was done on the 30 tumor and 26 adjacent normal oral tissue sample groups using the CLC genomics workbench to identify the differentially expressed genes between the two groups. A volcano plot representing this analysis was generated using RStudio 1.4.1106 (R version 4.0.5 (2021-03-31)). Additionally, a heat map was created to visualize the gene expression levels.

Subsequently the data obtained from the differential sequence analysis was filtered based on log2 fold change (> 2 or <-2) and FDR p-value (≤ 0.05) to identify significantly differentially expressed upregulated and downregulated genes.

Gene ontology (GO) analysis was done for the upregulated and downregulated genes using the Funrich (3.1.3) tool^19^ to identify the molecular function, biological process, biological pathway, cellular component and site of expression that these genes are associated with.

A protein-protein interaction (PPI) network was constructed for the upregulated and downregulated genes using the STRING database, and K-mean clustering was done to obtain three clusters. For each of these three clusters, the top 20 nodes were identified using the cytoHubba plugin in Cytoscape (3.10.0)^20^.

To identify the genes that are significant in HNSC, the top 100 upregulated and downregulated genes were validated using the TCGA (The Cancer genome atlas)^21^ database through the GEPIA2 tool^22^ (cutoffs log2 fold change (> 2 or <-2) and p-value (≤ 0.05)).

Box plots were made to observe and represent the differential expression between the tumor and adjacent normal sample groups for the upregulated and downregulated genes significant in HNSC using TPM values, and the significance values were calculated using the T-test.

Additionally, ROC (Receiver Operating Characteristic) curve analysis was done for the significant upregulated and downregulated genes through MetaboAnalyst (6.0) (last accessed on 5th december 2024). These genes were then sorted based on the highest to lowest AUC (area under the curve) value obtained from the ROC curves^23^.

To further study the trend in the expression levels of the significant genes, the significant upregulated and downregulated genes were also sorted based on highest to lowest log2 fold change and average tumor and adjacent normal RPKM (Reads Per Kilobase of transcript per Million mapped reads) separately.

#### Cancer stage-specific transcriptomic analysis

The dataset included 5 normal and 8 tumor samples (stage 1), 10 normal and 9 tumor (stage 2), 1 normal and 3 tumor (stage 3), and 8 normal and 7 tumor (stage 4) (Supplementary Fig. 5). Subsequent data analysis was done based on the stage of cancer, following steps similar to those outlined in section “Comprehensive transcriptomic analysis of the complete dataset”. Gene expression analysis was conducted by determining the number of upregulated and downregulated genes at each stage and examining their log2 fold changes.

#### Pairwise transcriptomic analysis of individual samples

The dataset included 20 true tumor and adjacent normal sample pairs. Data analysis was done for each individual sample pair, following steps similar to those outlined in section “Comprehensive transcriptomic analysis of the complete dataset”. Gene expression analysis was performed for each pair by identifying upregulated and downregulated genes and assessing their log2 fold changes.

#### Selection of significant differentially expressed genes

To identify key biomarker candidates for HNSC, genes were selected based on log2 fold change, average tumor/ adjacent normal RPKM, and AUC values from ROC curve analysis. Additionally differential expression was assessed using box plots, cancer stage comparisons, and individual sample analysis. This ensured the selection of genes with strong and consistent expression patterns for reliable biomarker potential.

### Experimental validation of selected differentially expressed genes via real time quantitative reverse transcription PCR (RT-qPCR)

Based on the gene expression analysis results, three genes *(MMP13*, *MMP10*, and *ADAM12*) were selected for RT-qPCR validation. Reverse transcription was performed using the PrimeScript™ RT reagent Kit with gDNA Eraser (Takara Biotechnology Co., Ltd.). TaqMan assays were conducted with specifically designed primers and probes using the Probe qPCR with UNG kit (Takara^®^), following the manufacturer’s protocol and at an annealing temperature of 60 °C. Reactions were performed on the Applied Biosystems 7500 Fast RT-PCR system, and data were analyzed using the comparative Ct (ΔΔCt) method with *GAPDH* as the control.

## Results

### Transcriptomic analysis

Transcriptomic analysis was done to identify significant differentially expressed genes that have the potential to serve as biomarkers to demarcate between tumor and non-tumor tissue.

The sequenced reads data for all the samples passed the quality check and were successfully mapped to the reference human genome (GRCh38.p13). Subsequently, the PCA plot generated for the 32 tumor and 27 adjacent normal samples in the CLC genomics workbench identified three outliers (Supplementary Fig. 1a), which were then excluded from the dataset (Supplementary Fig. 1b). All further data analysis was then performed on the remaining 30 tumor and 26 adjacent normal samples. The presence of outliers can cause discrepancies in data analysis, and their extreme values can influence the results, therefore eliminating outliers can increase accuracy in data analysis.

#### Comprehensive transcriptomic analysis of the complete dataset

**Fig. 1:**
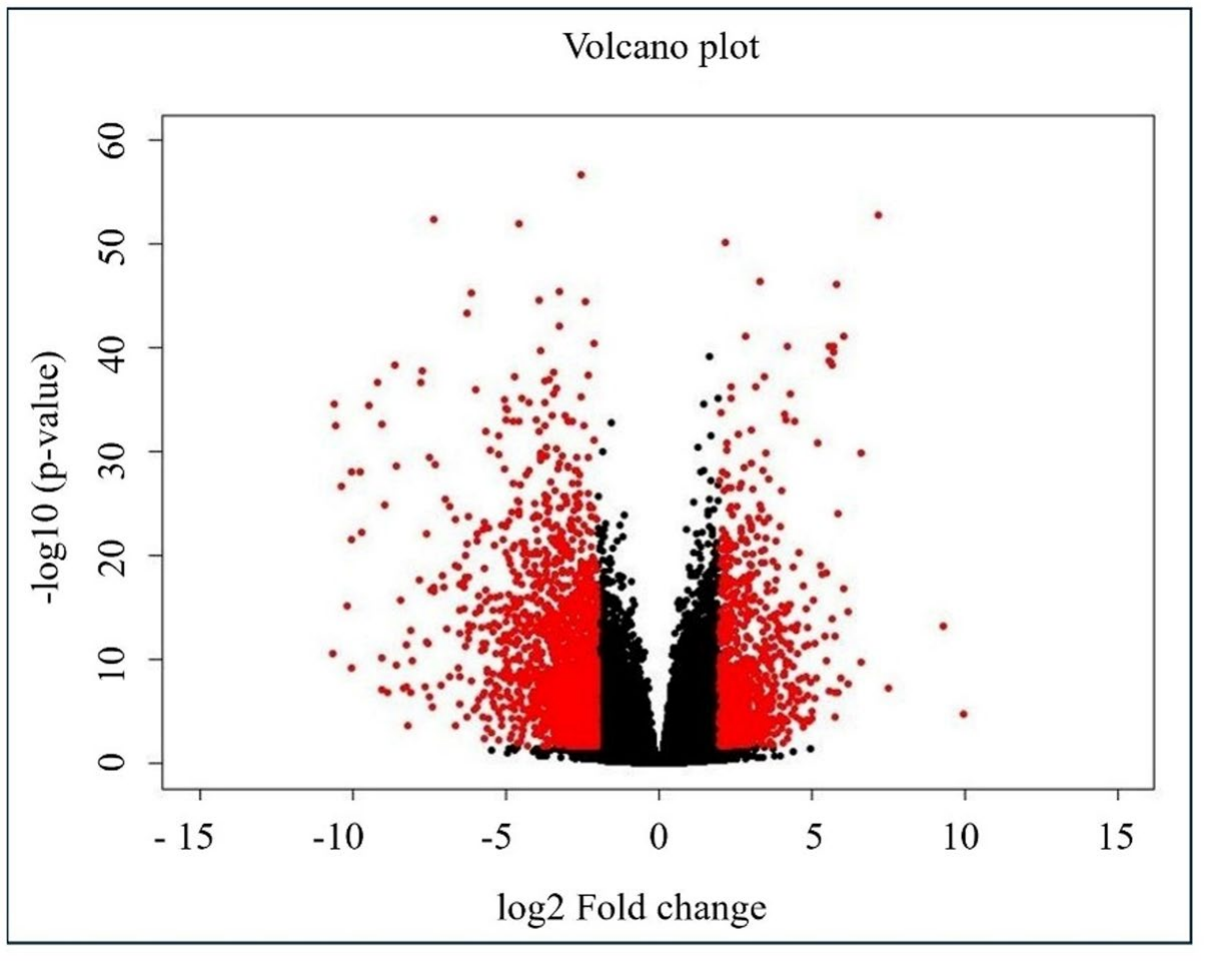
Volcano plot created through RStudio with the cutoffs of log2 fold change > 2 or <-2 and p-value as ≤ 0.05, representing the differentially expressed genes i.e. upregulated and downregulated genes (red dots).

Followed by mapping and PCA plot analysis, the differential sequence analysis conducted on 30 tumor and 26 adjacent normal sample, identified 704 genes as upregulated and 1540 genes as downregulated using the criteria of log2 fold change (> 2 or <-2) and FDR p-value (≤ 0.05). These results are graphically represented by a volcano plot (Fig. 1). Additionally, gene expression levels across all tumor and adjacent normal samples were visualized using a heat map as it shows the relative intensity of the expression values (Supplementary Fig. 2).

**Fig. 2:**
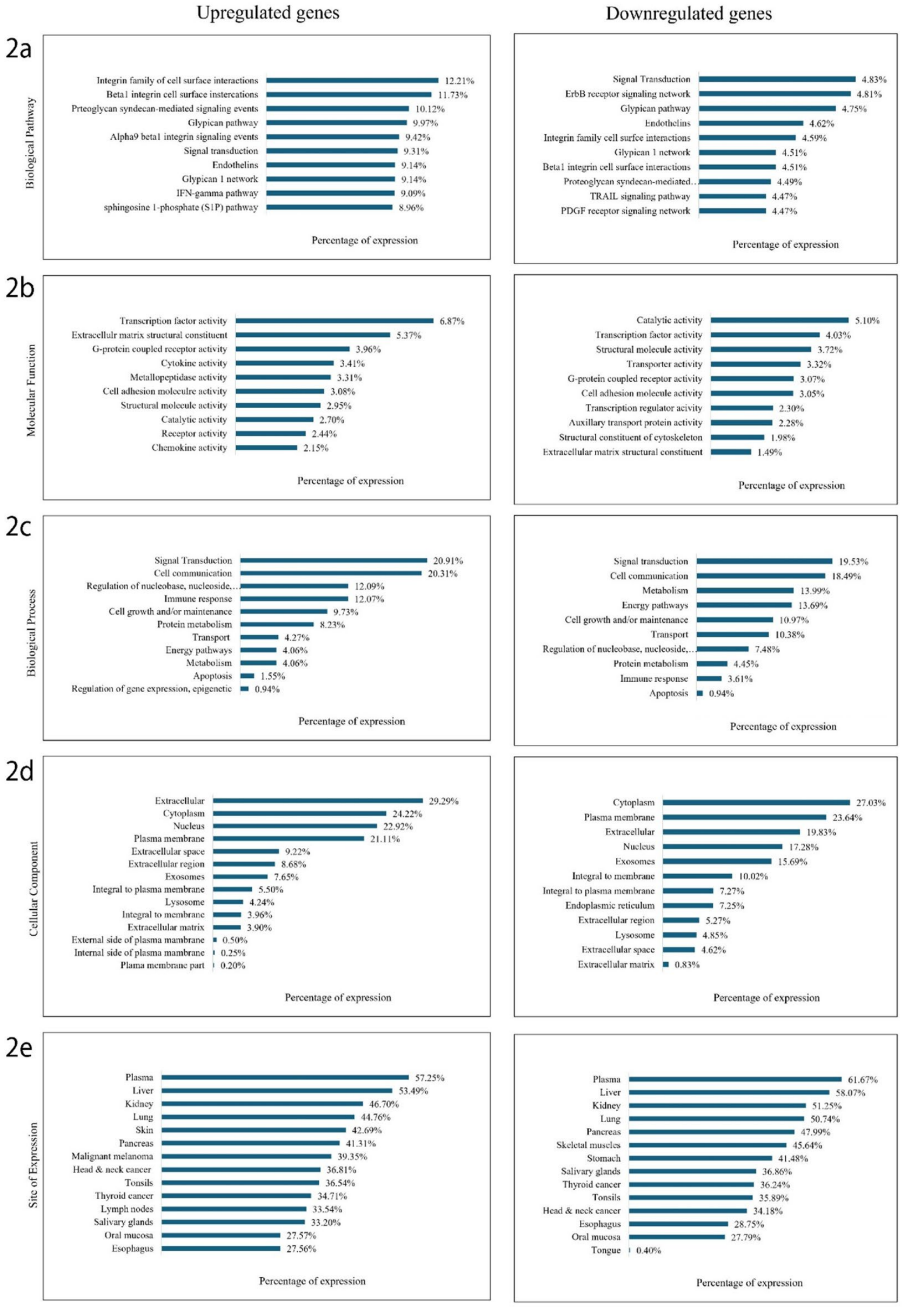
Gene ontology (GO) analysis of upregulated and downregulated genes in tumour and adjacent normal tissue identified using cutoff thresholds of log2 fold change > 2 or < -2 and p-value ≤ 0.05. The graphs represent the percentage of gene expression across various GO categories: (**a**) Biological pathways, (**b**) Molecular functions, (**c**) Biological processes, (**d**) Cellular components, and (**e**) Expression sites.

Further the gene ontology analysis of these upregulated and downregulated genes identified that the greatest percentage of upregulated genes were associated with biological pathways like the Integrin family of cell surface interactions (Fig. 2a). Additionally, the highest percentage of downregulated genes were associated with catalytic activity molecular function while the greatest percentage of upregulated genes were associated with transcription factor molecular function (Fig. 2b). In association to biological processes, the highest percentage of upregulated and downregulated genes were correlated with Signal transduction (Fig. 2c). Moreover, a high percentage of upregulated and downregulated genes were found to be extracellular and present in the cytoplasm, nucleus and plasma membrane cellular components (Fig. 2d). Further when analyzed for site of expression for the upregulated and downregulated genes, a high percentage of expression was observed for Head and neck cancer (Fig. 2e).

The PPI network created for the upregulated and downregulated genes followed by clustering resulted in 3 clusters (Supplementary Fig. 3a, 4a). For the upregulated genes, cluster 1 contained 9 out of the 15 identified genes significant in HNSC, while cluster 2 and 3 contained 5 and 1 genes respectively. Whereas for downregulated genes, cluster 1 contained 7 out of the 9 identified genes significant in HNSC and cluster 2 contained 2 genes. The PPI networks represent the physical interactions between proteins, while K-means clustering is used to group datasets into distinct clusters based on similarity. For each identified cluster, the top 20 nodes were analyzed using CytoHubba, which revealed that cluster 1 of upregulated genes and cluster 3 of downregulated genes contained a significant number of genes associated with HNSC (Supplementary Fig. 3b, 4b). Cytohubba aids in predicting and exploring significant nodes in a network and is used to identify hub genes.

**Fig. 3:**
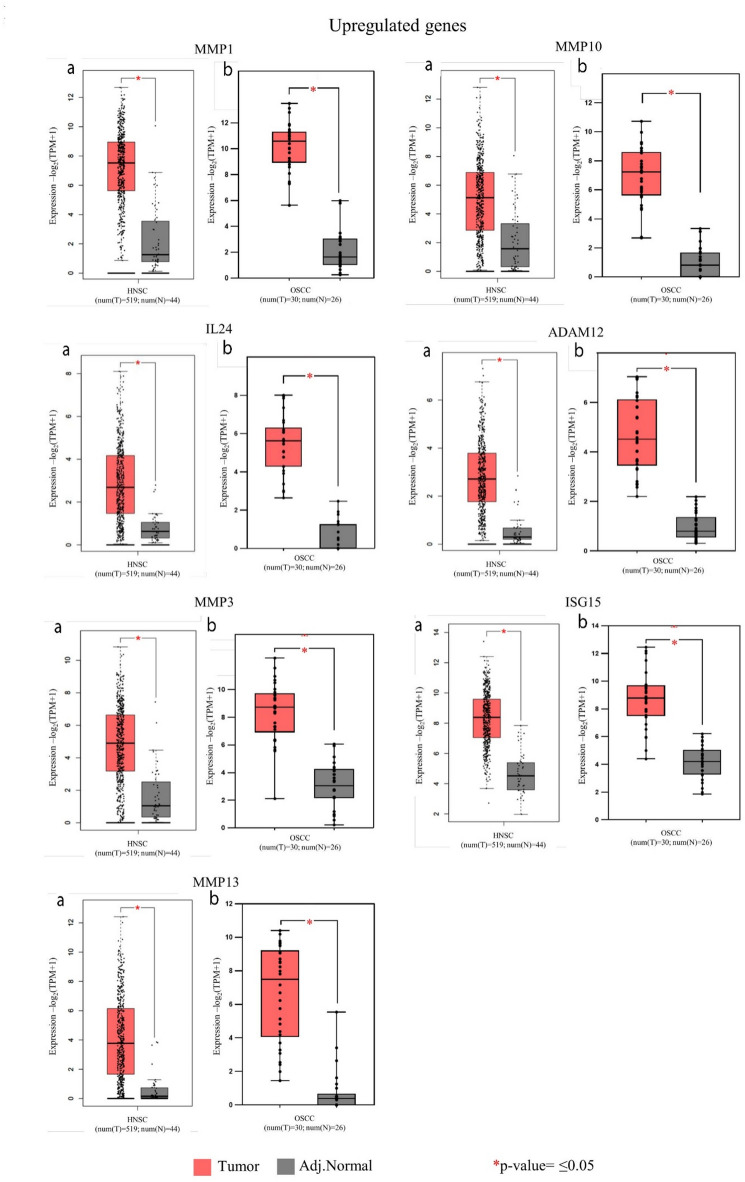
Representative box plot showing differential upregulated gene expression between tumor and adjacent normal tissue with p-value ≤ 0.05. For each of the significant upregulated genes, box plots labelled ‘**a**’ are of TCGA database and box plots labelled ‘**b**’ are of the data from the current study.

**Fig. 4:**
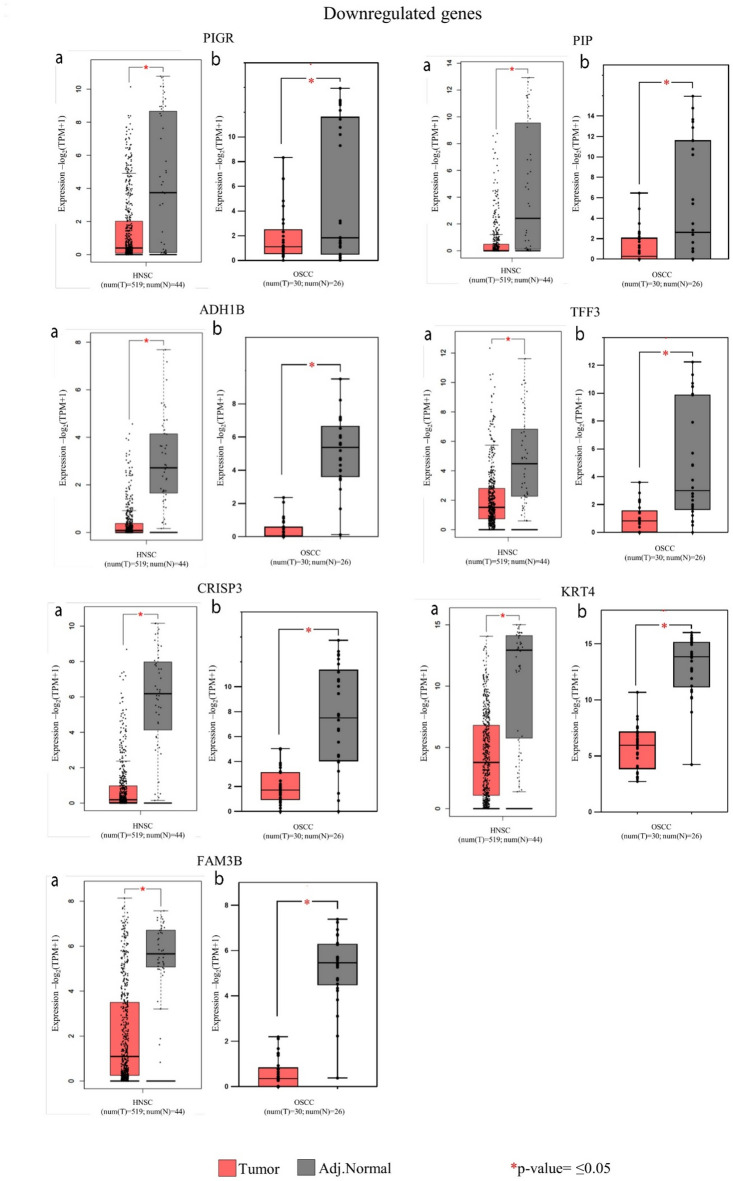
Representative box plot showing differential downregulated gene expression between tumor and adjacent normal tissue with p-value ≤ 0.05. For each of the significant downregulated genes, box plots labelled ‘**a**’ are of TCGA database and box plots labelled ‘**b**’ are of the data from the current study.

Furthermore, the validation of the identified top 100 upregulated and downregulated genes, performed using the TCGA database through the GEPIA2 tool (Supplementary table. 2a, 2b), showed that 15 upregulated and 9 downregulated genes were significant in HNSC (Table 1). These findings were illustrated through box plot representations (Figs. 3 and 4). Notably, HNSC was chosen for validation studies as GEPIA and TCGA databases categorize data under HNSC instead of OSCC, which accounts for 90% of HNSC cases. The TCGA database, a significant cancer genomics initiative, contains more than 20,000 cancer and matched normal samples from 33 different cancer types. Similarly, the GEPIA2 tool, used for analysing sequencing expression data, contains 9736 tumor and 8537 normal samples data from TCGA and GTEX projects.

The box plots for the 15 upregulated (Fig. 3) and 9 downregulated (Fig. 4) genes that were significant in HNSC showed substantial differential expression between the tumor and adjacent normal groups. Significance calculated using the T- test showed that all these genes were significant with a p-value ≤ 0.05 (Figs. 3 and 4).

**Fig. 5:**
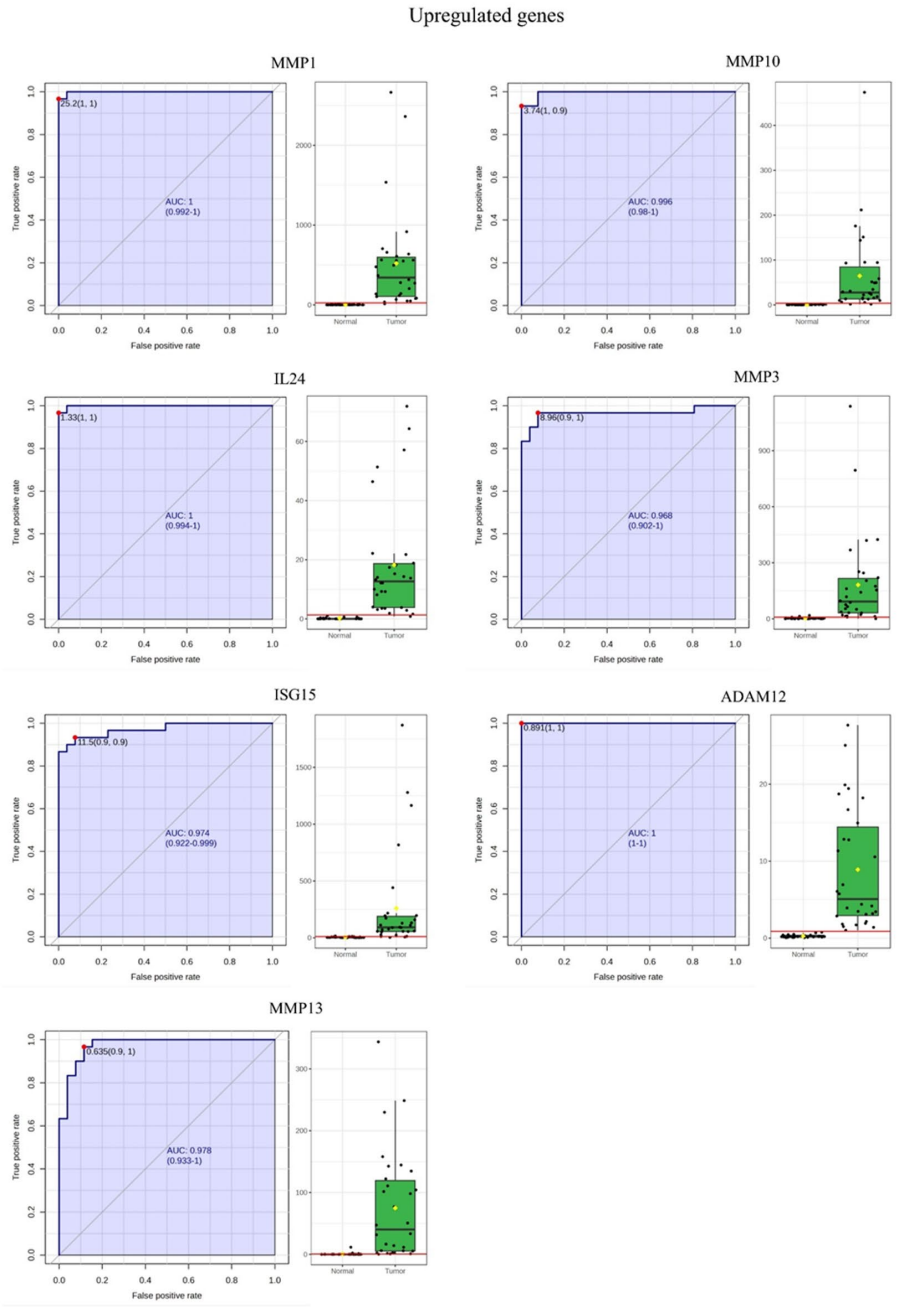

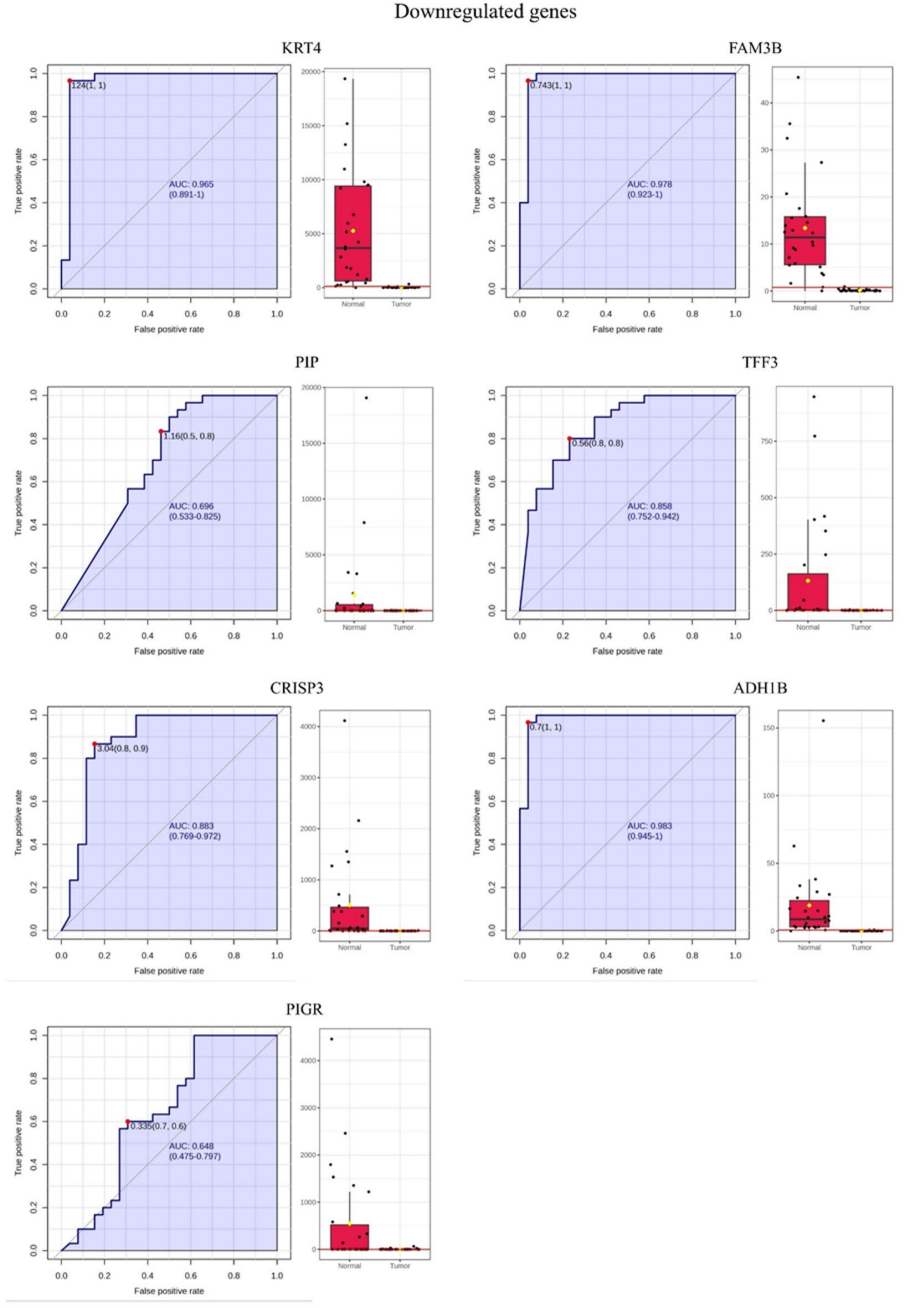
Representative ROC curves of the significant upregulated and downregulated genes in tumor and adjacent normal tissues and their AUC values.

Subsequently, the ROC curves generated for the significant upregulated and downregulated genes revealed that most had an AUC closer to 1 while the genes *MMP1*, *IL24* and *ADAM12* achieved a perfect AUC of 1 (Table 1). Representative ROC curves for the significant upregulated and downregulated genes are shown in Fig. 5. ROC curve analysis is generally considered the standard method for performance assessment in biomarker studies and the closer the AUC is to 1, the better is the utility of the biomarker. An AUC value of 1, or closer to it, indicates a high true positive rate and a low false positive rate.

**Table 1:** The table presents a list of 15 upregulated and 9 downregulated genes, identified as significant in HNSC and ranked within the top 100 upregulated and downregulated genes. The upregulated genes *MMP1*, *MMP10*, *IL24*, *MMP13* and downregulated genes *PIP*, *TFF3*, *CRISP3* had the highest log2 fold change, the upregulated genes *MMP1*, *IL24*, *ADAM12*, *MMP10* and downregulated genes *ADH1B*, *FAM3B*, *KRT4* had the highest AUC values, and the upregulated genes *MMP1*, *ISG15*, *MMP3*, *MMP13* and downregulated genes *KRT4*, *PIP*, *PIGR* showed the highest RPKM levels (highlighted in bold).

		Name	Log2 fold change	FDR *p*-value	ROC curve value	Avg. Tumor RPKM	Avg. Normal RPKM
Upregulated genes significant in HNSC	1	*MMP1*	**7.904**	0	**1**	**520.547**	1.795
	2	*MMP10*	**7.254**	0	**0.996**	64.527	0.362
	3	*IL24*	**6.694**	0	**1**	18.206	0.151
	4	*MMP13*	**6.676**	0	0.978	**74.946**	0.647
	5	*IL11*	6.163	0	0.964	3.952	0.039
	6	*CSAG3*	5.758	0	0.895	2.353	0.029
	7	*MMP3*	5.567	0	0.969	**181.501**	3.319
	8	*ISG15*	5.548	0	0.976	**259.293**	5.170
	9	*CA9*	5.385	0	0.849	2.328	0.049
	10	*MMP12*	5.210	0	0.976	71.452	1.661
	11	*DNAH17*	5.043	0	0.987	2.084	0.057
	12	*ADAM12*	4.900	0	**1**	8.897	0.255
	13	*CXCL11*	4.561	0	0.907	37.445	1.234
	14	*S100A7A*	4.455	0	0.965	23.727	1.067
	15	*KHDC1L*	4.426	0	0.978	3.978	0.162
Downregulated genes significant in HNSC	1	*PIP*	**-10.546**	0	0.7	1.301	**1426.734**
	2	*TFF3*	**-8.626**	0	0.86	0.381	131.549
	3	*CRISP3*	**-8.597**	0	0.887	1.430	504.455
	4	*SCGB3A1*	-7.991	0	0.342	0.224	52.109
	5	*PPP1R1B*	-7.888	0	0.95	0.054	11.291
	6	*ADH1B*	-7.687	0	**0.983**	0.099	18.877
	7	*PIGR*	-7.395	0	0.648	4.396	**543.606**
	8	*KRT4*	-7.326	0	**0.965**	31.518	**5263.881**
	9	*FAM3B*	-6.505	0	**0.977**	0.164	13.387

To further examine the expression trends of significant genes, upregulated and downregulated genes were identified based on their log2 fold change, average tumor/adjacent normal RPKM, and AUC values. The results revealed that the upregulated genes *MMP1*, *MMP10*, *IL24*, *MMP3*, *ISG15*, *ADAM12*, *MMP13* and the downregulated genes *PIP*, *TFF3*, *CRISP3*, *ADH1B*, *PIGR*, *KRT4*, *FAM3B* had the highest log2 fold change, AUC value and avg. tumor/ adjacent normal RPKM (Table 1).

#### Cancer stage-specific transcriptomic analysis

The total number of upregulated and downregulated genes for each of the four stages has been mentioned in the Supplementary table. 3a. Additionally, when examining the log2 fold change of the significant upregulated and downregulated genes for each of the four stages, all of the upregulated genes were found to have a significant log2 fold change in all four cancer stages and the downregulated genes exhibited significant log2 fold change across most cancer stages (Supplementary table. 3b).

#### Pairwise transcriptomic analysis of individual samples

The number of upregulated and downregulated genes for each of the 20 sample pairs has been mentioned in the Supplementary table. 4a. Furthermore, upon studying the log2 fold change of the significant upregulated and downregulated genes for each of the 20 sample pairs, many of these upregulated and downregulated genes showed significant log2 fold changes across most individual sample pairs (Supplementary table. 4b).

#### Selection of significant differentially expressed genes

The upregulated genes were selected as potential biomarkers and the selection was based on the key parameters. *MMP1* emerged as a standout candidate with the highest log2 fold change, average tumor RPKM, and AUC value. Similarly, *MMP10* and *IL24* showed both one of the highest log2 fold change and AUC value. *MMP13* was notable for its high log2 fold change and average tumor RPKM. *ADAM12* displayed the highest AUC value, while *MMP3* and *ISG15* demonstrated one of the highest average tumor RPKM. Additionally, as observed from the box plots (Figs. 3 and 4), these genes also demonstrated significant differential expression between the tumor and adjacent normal groups. Along with this, they also showed significant log2 fold change across all four cancer stages and most individual sample pairs, as detailed in the analysis based on cancer stage and sample pairs (Supplementary table. 3b, 4b). Collectively, these genes represent strong candidates for use as tumor-specific potential biomarkers.

### Experimental validation of selected differentially expressed genes via real time quantitative reverse transcription PCR (RT-qPCR)

**Fig. 6:**
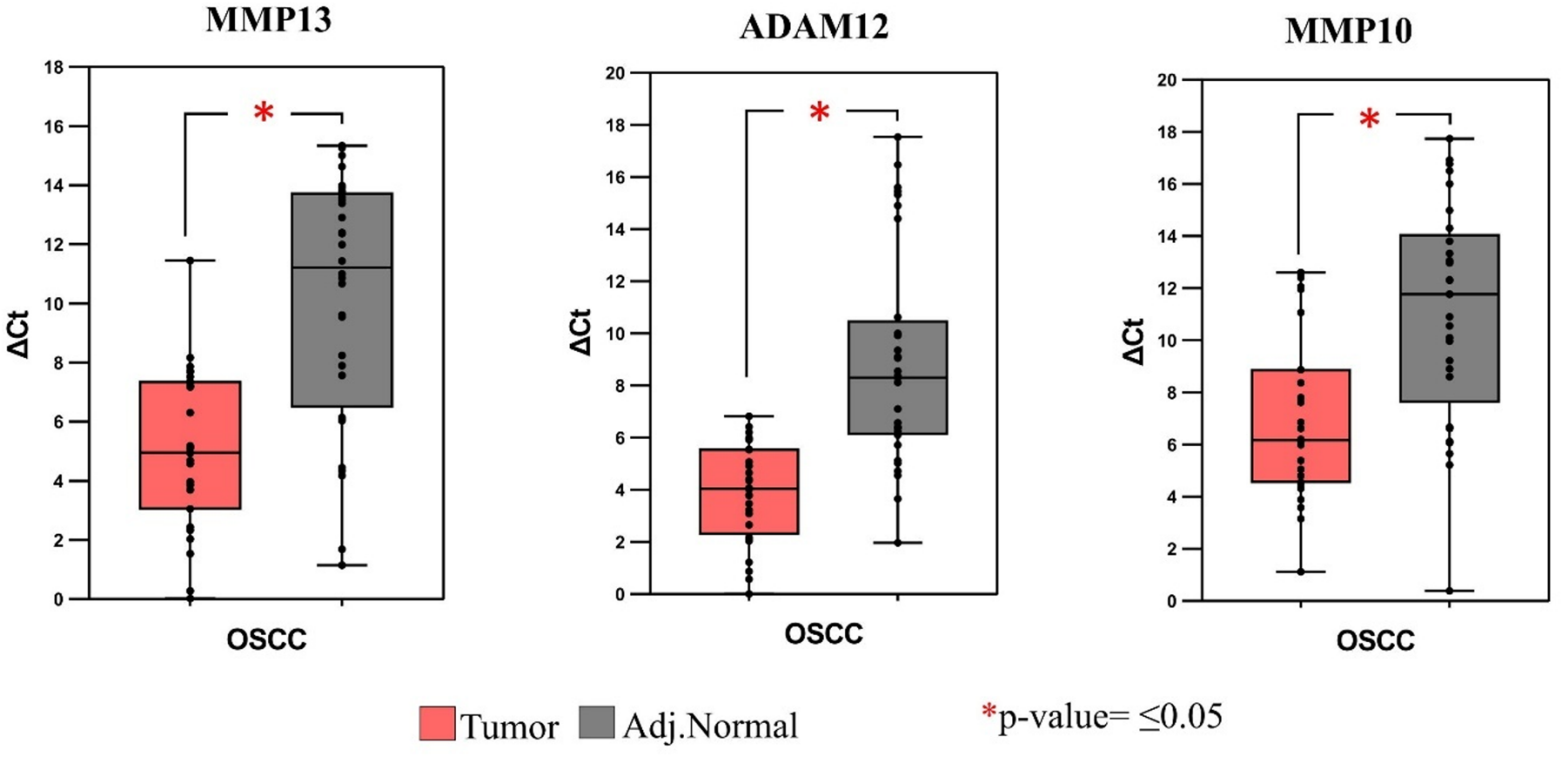
Box plots representing differential expression of *MMP13*, *MMP10* and *ADAM12* genes based on RT-qPCR data with the p-value of ≤ 0.05.

Significant differences in the expression levels of *MMP13*, *MMP10*, and *ADAM12* were observed between tumor and adjacent normal tissue samples, as illustrated in the box plots (Fig. 6). These differences were statistically significant, with *p* ≤ 0.05, as determined by the T-test. Furthermore, a comparison between the log₂ fold changes obtained from transcriptome analysis and RT-qPCR data demonstrated consistent and significant expression trends for all three genes. Specifically, RT-qPCR revealed log₂ fold changes of 5.08 for *MMP13*, 5.04 for *ADAM12*, and 3.99 for *MMP10*, while transcriptomic analysis showed corresponding values of 6.68, 4.90, and 7.25, respectively.

## Discussion

Based on the above findings, we further explored the study’s relevance and the biological implications of the identified significant genes as potential biomarkers. Numerous studies have focused on transcriptional dysregulation and disease-specific signatures in oral squamous cell carcinoma (OSCC) to explore carcinogenesis, prognostic indicators, and potential molecular targets^24^. Incomplete surgical excision of the primary tumor is a key factor affecting patient survival^25^. When recurrences are treated surgically, challenges arise due to the potential spread of microscopic tumor cells beneath normal mucosa or at a distance from the initial tumor site, complicating the determination of clear surgical margins^26^.

In this study we identified significant differentially expressed genes that can serve as potential molecular biomarkers for demarcating tumor from non-tumor tissues. This is critical because tissue beyond clinically determined margins may appear morphologically normal yet possess molecular alterations characteristic of OSCC, contributing to recurrence, which remains a major challenge. The biomarkers identified in this study by tumor versus adjacent normal comparison could support more effective margin determination by enabling molecular assessment of tissues beyond the planned surgical boundary. If these markers show elevated expression in “normal appearing” tissue, the surgical margin could be adjusted accordingly.

Through differential expression analysis we identified the 704 upregulated and 1540 downregulated genes. Upon gene ontology analysis it was revealed that the greatest percentage of upregulated genes were associated with biological pathways like the Integrin family of cell surface interactions (Fig. 2a). Integrins are cell surface receptors and transmembrane plasma membrane proteins. They are made of α- and β-chains and activate focal adhesion kinase (FAK) upon ECM (extracellular matrix) protein binding. A study showed the expression of αV, β1, β3, β5, β6, FAK and pFAK (phosphorylated-FAK) integrins as prognostic predictors in OSCC patients. It highlighted that the pFAK-positive OSCC patients had a decreased overall survival rate as compared to the negative group. A study identified αV, β1, β3, β5, β6, FAK, and pFAK integrins as prognostic markers in OSCC. pFAK-positive patients had lower overall survival, highlighting integrin β8 and pFAK as potential diagnostic and therapeutic targets for oral cancer^27^. In OSCC, there is a loss or reduction expression of alpha 6 beta 4 integrins and de novo expression of alpha v beta 6 integrins which relates to basement membrane protein loss and may be essential in tumor cell migration respectively^28^. In association to biological processes, the highest percentage of upregulated and downregulated genes were correlated with Signal transduction (Fig. 2c). In the progression and development of cancer, cell signal transduction is a fundamental process. Several key signal transduction pathways are dysregulated in cancer cells, including NF-κB/nuclear factor kappa beta, *p53*, protein kinase B/AKT, mammalian target of rapamycin/mTOR, β-catenin, c-Myc^29^, and JAK/STAT pathways^30^. STAT3 is involved in key aspects of cancer metastasis, such as invasion, migration, and angiogenesis^31^. A critical step in the metastatic process is the ability of cancer cells to invade the extracellular matrix, a process facilitated by the regulation of matrix metalloproteinases *(MMP*s)^32^. Also, the IL-6/STAT3 signalling pathway enhances the expression of *MMP*s such as *MMP1*, *MMP2*, *MMP7*, and *MMP9* by directly binding to their promoter regions, a mechanism observed in various aggressive cancer types^33^.

Additionally, the highest percentage of downregulated genes were associated with catalytic activity molecular function while the greatest percentage of upregulated genes were associated with transcription factor molecular function (Fig. 2b). Transcription factors (TFs) are proteins that control gene expression and are regularly varied in oral cancer. Some TFs are correlated with oral cancer such as overexpression of c-myc^34^, c-Jun and Fra-1^35^ are associated with poor prognosis and poorly differentiated tumors, abnormal activation and expression of *TEAD* is correlated to development of OSCC^36^ and low expression of *FOXO1* and *HBP1* is linked with oral tumor invasiveness^37^. In context to cellular component, highest percentage of upregulated and downregulated genes were found to be extracellular and present in the cytoplasm, nucleus and plasma membrane (Fig. 2d). This enhances their detectability, diagnostic relevance and facilitates in identifying druggable targets especially extracellular and membrane proteins^38^. Further when analyzed for site of expression for the upregulated and downregulated genes, a high percentage of expression was observed for Head and neck cancer (Fig. 2e).

We further identified genes significant in HNSC from the top 100 upregulated and downregulated genes and selected those with the highest potential as effective biomarkers for demarcating tumor tissues from non-tumor tissues to facilitate efficient margin clearance. This analysis highlighted *MMP1*, *MMP10*, *MMP13*, *MMP3*, *ADAM12*, *IL24*, and *ISG15* as key candidates. These genes, exhibiting significantly elevated expression in oral tumor samples, are promising detectable biomarkers indicative of tumor presence, particularly those localized to the cell’s outer regions.

Furthermore, gaining insights into the functions of these significantly upregulated genes is essential for understanding the implications of their deregulation. Significantly upregulated genes like *IL24*, *ISG15* and *ADAM12* have been found to play a significant role in oral cancer. *ADAM12* (ADAM Metallopeptidase Domain 12) that is a part of metalloproteinase and disintegrin family of membrane related metalloproteinases was found to have one of the highest AUC values (1.0) suggesting its significant upregulation in oral tumor samples and its potential to be an effective biomarker in HNSC studies. As supported by findings from other studies, *ADAM12* is associated with numerous malignant tumors. Its overexpression in OSCC has been shown to enhance cellular proliferation, metastasis, and invasion, suggesting its potential role as an oncogene^39^ and its function may also be related with TGF-β signalling^40^. Whereas *IL24* (Interleukin-24) with one of the highest log2 fold change (6.69) and AUC value (1.0) in this study (Table 1) has been found to induce apoptosis in cancer cells with minimal effect on normal cells in previous studies. It has also been suggested that by inhibiting tumor invasion, growth and angiogenesis, higher expression of *IL24* can be correlated with better prognosis in OSCC, and it can be a capable target for cancer therapy^41^. Moreover, studies have indicated that *ISG15* (Interferon-stimulated gene 15) is often upregulated in oral cancer and contributes to cancer cell invasion, migration and metastasis playing a pro-tumorigenic role. This correlates to poor prognosis in OSCC and can be considered a possible biomarker for disease progression^42^. Even in this study *ISG15* was found to have one of the highest tumor RPKM values (Table 1). Furthermore, Matrix Metallopeptidase genes such as *MMP1*, *MMP10*, *MMP13* and *MMP3* that encode a member of the peptidase M10 family of matrix metalloproteinases (*MMP*s) showed significant upregulation in the oral tumor samples (Table 1). *MMP1* especially emerged as a standout candidate with the highest log2 fold change (7.90), average tumor RPKM (520.55), and AUC value (1.0) among all the identified significantly upregulated genes (Table 1). Other studies have shown that *MMP*s are overexpressed in HNSC, and their expression has been associated with tumorigenic hallmarks like cell proliferation, metastasis, angiogenesis, and invasion^43^. *MMP10* plays a crucial role in metastasis and invasion of HNSC and is partly associated with the *p38* MAPK inhibition^44^ and can be used as a marker for metastasis prediction. It is expressed in tissues of OSCC and can serve as prognostic indicators^45^. Moreover, the overexpression of *MMP1* and *MMP13* has been associated with the aggressive behaviour of OSCC, facilitating the invasion of nearby bone in patients with OSCC^46^. Also, the presence of *MMP1* and *MMP3* in saliva could serve as a non-invasive biomarker for the early detection of oral cancer^47^. Additionally, through GO analysis it was identified that *MMP1* along with other *MMP*s was mainly associated with molecular function of metallopeptidase activity, biological pathway of Beta1 integrin cell surface interactions and protein metabolism biological process (Fig. 2). This study highlights the significantly upregulated genes *MMP1*, *MMP10*, *MMP13*, *MMP3*, *ADAM12*, *IL24*, and *ISG15* as potential molecular biomarkers for demarcating tumor from non-tumor tissues, thereby improving the efficiency of margin clearance. These genes demonstrated the highest log2 fold change, tumor RPKM, AUC values and significant differences in expression levels as validated through RT-qPCR, underlining their relevance for assessing surgical margins at the molecular level.

However, validation in actual surgical margin specimens or clinically relevant borderline tissues has not yet been performed, primarily due to their limited access. Additionally, the study does not yet provide direct evidence supporting translation of these findings into actual clinical application, including the development of rapid intraoperative molecular assays.

Future studies in the field could therefore address these gaps by validating these biomarkers at the protein level, assessing their performance directly in surgical margin and borderline tissues, and developing rapid, clinically applicable detection methods that can advance their translation toward practical intraoperative utility.

## Conclusion

OSCC is a prevalent and challenging malignancy, especially in India, with high recurrence despite treatment advances. This study highlights the need for reliable biomarkers to demarcate tumor from non-tumor tissue. Through transcriptomic analysis, key differentially expressed genes (DEGs) were identified, including *MMP1*, *MMP10*, *MMP13*, *MMP3*, *ADAM12*, *IL24*, and *ISG15* that showed the strongest potential for accurate tissue demarcation. These findings with further exploration pave the way for clinically applicable tools to potentially improve surgical margin evaluation, reduce recurrence rates, and enhance patient outcomes, supporting precision surgery in OSCC treatment.

## Supplementary Information

Below is the link to the electronic supplementary material.

Supplementary Material 1

## Data Availability

The datasets generated and/or analysed during the current study are available in the NCBI repository, under BioProject PRJNA1127288 (https://www.ncbi.nlm.nih.gov/bioproject/?term=PRJNA1127288).All data generated or analysed during this study are included in this published article and its supplementary information files.

## References

[1] Singh, P. et al. Survival-based biomarker module identification associated with oral squamous cell carcinoma (Oscc). *Biology (Basel)* **10**, 760 (2021). 10.3390/biology10080760 PMC8389591 34439992

[2] Maheswaran Easwaran, S. M. Theranostic potential of bacteriophages against oral squamous cell carcinoma. *Curr. Gene Ther.* **25**, 89–91 (2024). 10.2174/0115665232305905240521081553 38808710

[3] GLOBOCAN. (2022). https://gco.iarc.who.int/media/globocan/factsheets/populations/900-world-fact-sheet.pdf

[4] GLOBOCAN. (2022). https://gco.iarc.who.int/media/globocan/factsheets/populations/356-india-fact-sheet.pdf

[5] Gupta, B., Bray, F., Kumar, N. & Johnson, N. W. Associations between oral hygiene habits, diet, tobacco and alcohol and risk of oral cancer: A case–control study from India. *Cancer Epidemiol.* **51**, 7–14 (2017). 28968558 10.1016/j.canep.2017.09.003

[6] Subash, A., Bylapudi, B., Thakur, S. & Rao, V. U. S. Oral cancer in India, a growing problem: is limiting the exposure to avoidable risk factors the only way to reduce the disease burden? *Oral Oncol.* **125**, 105677 (2022). 34954504 10.1016/j.oraloncology.2021.105677

[7] Johnson, D. E. et al. Head and neck squamous cell carcinoma. *Nat. Rev. Dis. Prim.* **6**, 92 (2020). 10.1038/s41572-020-00224-3 PMC7944998 33243986

[8] Ameya, K. P., Ashikha Shirin Usman, P. P. & Sekar, D. OIP5-AS1 expression profiles in different stages of oral squamous cell carcinoma. Arch. Oral Biol. **180**, 106403 (2025). 10.1016/j.archoralbio.2025.106403 41014896

[9] Ferlay, J. et al. Estimating the global cancer incidence and mortality in 2018: GLOBOCAN sources and methods. *Int. J. Cancer*. **144**, 1941–1953 (2019). 30350310 10.1002/ijc.31937

[10] Tapak, L. et al. Identification of gene profiles related to the development of oral cancer using a deep learning technique. *BMC Med. Genomics*. **16**, 1–12 (2023). 36849997 10.1186/s12920-023-01462-6 PMC9972685

[11] Xing, A. et al. Tertiary lymphoid structures gene signature predicts prognosis and immune infiltration analysis in head and neck squamous cell carcinoma. *Curr. Genom.* **25**, 88–104 (2024). 10.2174/0113892029278082240118053857 PMC11092909 38751598

[12] Grafton-Clarke, C., Chen, K. W. & Wilcock, J. Diagnosis and referral delays in primary care for oral squamous cell cancer: A systematic review. *Br. J. Gen. Pract.* **69**, E112–E126 (2019). 30455220 10.3399/bjgp18X700205 PMC6355296

[13] Chen, T. C. et al. The clinical predictive factors for subsequent distant metastasis in patients with locoregionally advanced oral squamous cell carcinoma. *Oral Oncol.* **49**, 367–373 (2013). 23142556 10.1016/j.oraloncology.2012.10.006

[14] Jiang, W. & Xu, S. SLC2A3 is a potential factor for head and neck squamous cancer development through tumor microenvironment alteration. *Curr. Gene Ther.* **25**, 157–177 (2025). 38778609 10.2174/0115665232291300240509104344 PMC11774314

[15] Chen, C. et al. Gene expression profiling identifies genes predictive of oral squamous cell carcinoma. *Cancer Epidemiol. Biomarkers Prev.* **17**, 2152–2162 (2008). 18669583 10.1158/1055-9965.EPI-07-2893 PMC2575803

[16] Lin, M. C. et al. Adequate surgical margins for oral cancer: A Taiwan cancer registry National database analysis. *Oral Oncol.* **119**, 105358 (2021). 34049257 10.1016/j.oraloncology.2021.105358

[17] Barroso, E. M. et al. Performance of intraoperative assessment of resection margins in oral cancer surgery: A review of literature. *Front. Oncol.* **11**, 1–10 (2021). 10.3389/fonc.2021.628297 PMC8044914 33869013

[18] Kang, C. J. et al. Surgical margins status and prognosis after resection of oral cavity squamous cell carcinoma: results from a Taiwanese nationwide registry-based study. *Cancers (Basel)* **14**, 15 (2022). 10.3390/cancers14010015 PMC8749941 35008181

[19] Fonseka, P., Pathan, M., Chitti, S. V., Kang, T. & Mathivanan, S. FunRich enables enrichment analysis of omics datasets. *J. Mol. Biol.* **433**, 166747 (2021). 33310018 10.1016/j.jmb.2020.166747

[20] Paul Shannon, 1 et al. Cytoscape: A software environment for integrated models. *Genome Res.* **13**, 426 (1971). 10.1101/gr.1239303 PMC403769 14597658

[21] Lawrence, M. S. et al. Comprehensive genomic characterization of head and neck squamous cell carcinomas. *Nature* **517**, 576–582 (2015). 25631445 10.1038/nature14129 PMC4311405

[22] Tang, Z., Kang, B., Li, C., Chen, T. & Zhang, Z. GEPIA2: an enhanced web server for large-scale expression profiling and interactive analysis. *Nucleic Acids Res.* **47**, W556–W560 (2019). 31114875 10.1093/nar/gkz430 PMC6602440

[23] Xia, J., Broadhurst, D. I., Wilson, M. & Wishart, D. S. Translational biomarker discovery in clinical metabolomics: an introductory tutorial. *Metabolomics* **9**, 280–299 (2013). 23543913 10.1007/s11306-012-0482-9 PMC3608878

[24] Ziober, A. F. et al. Identification of a gene signature for rapid screening of oral squamous cell carcinoma. *Clin. Cancer Res.* **12**, 5960–5971 (2006). 17062667 10.1158/1078-0432.CCR-06-0535

[25] Fox, S. A., Vacher, M. & Farah, C. S. Transcriptomic biomarker signatures for discrimination of oral cancer surgical margins. *Biomolecules* **12**, 464 (2022). 10.3390/biom12030464 PMC8946245 35327656

[26] Thomas Robbins, K. et al. Surgical margins in head and neck cancer: Intra- and postoperative considerations. *Auris Nasus Larynx*. **46**, 10–17 (2019). 30172560 10.1016/j.anl.2018.08.011

[27] Sakurai, S. et al. Clinical significance of integrin αV and β superfamily members and focal adhesion kinase activity in oral squamous cell carcinoma: a retrospective observational study. *Pathol. Oncol. Res.* **30**, 1–11 (2024). 10.3389/pore.2024.1611571 PMC10830843 38312516

[28] Thomas, G., Jones, J. & Speight, J. Reviews integrins and oral cancer. *Oral Oncol.* **33**, 381–388 (2000). 10.1016/s0964-1955(97)00021-3 9509120

[29] Lakshminarayana, S. et al. Molecular pathways of oral cancer that predict prognosis and survival: A systematic review. *J. Carcinog.* **17**, 7 (2018). 30766450 10.4103/jcar.JCar_17_18 PMC6334533

[30] Choi, H. S., Kim, Y. K. & Yun, P. Y. Upregulation of mdr- and emt-related molecules in cisplatin-resistant human oral squamous cell carcinoma cell lines. *Int. J. Mol. Sci.* **20**, 3034 (2019). 10.3390/ijms20123034 PMC6627081 31234332

[31] Thomas, P., Selvakumar, S. C., Preethi, K. A. & Sekar, D. Expression profiling of signal transducer and activator of transcription3 in oral squamous cell carcinoma in south Indian population. *Minerva Dent. Oral Sci.* **73**, 37–44 (2024). 10.23736/S2724-6329.23.04840-4 37878241

[32] Verma, R. P. & Hansch, C. Matrix metalloproteinases (MMPs): Chemical-biological functions and (Q)SARs. *Bioorg. Med. Chem.* **15**, 2223–2268 (2007). 17275314 10.1016/j.bmc.2007.01.011

[33] Zugowski, C. et al. STAT3 controls matrix metalloproteinase-1 expression in colon carcinoma cells by both direct and AP-1-mediated interaction with the MMP-1 promoter. *Biol. Chem.* **392**, 449–459 (2011). 21410405 10.1515/BC.2011.038

[34] Jurel, S. K., Gupta, D. S., Singh, R. D., Singh, M. & Srivastava, S. Genes and oral cancer. *Indian J. Hum. Genet.* **20**, 4–9 (2014). 24959008 10.4103/0971-6866.132745 PMC4065477

[35] Xu, H. et al. Prognostic value from integrative analysis of transcription factors c-Jun and Fra-1 in oral squamous cell carcinoma: A multicenter cohort study. *Sci. Rep.* **7**, 1–9 (2017). 28790303 10.1038/s41598-017-05106-5 PMC5548725

[36] Wang, S. et al. TEAD transcription factor family emerges as a promising therapeutic target for oral squamous cell carcinoma. *Front. Immunol.* **15**, 1480701 (2024). 39430767 10.3389/fimmu.2024.1480701 PMC11486717

[37] Chan, C. Y. et al. Transcription factor HBP1 is a direct anti-cancer target of transcription factor FOXO1 in invasive oral cancer. *Oncotarget* **8**, 14537–14548 (2017). 28099936 10.18632/oncotarget.14653 PMC5362424

[38] Leth-Larsen, R., Lund, R. R. & Ditzel, H. J. Plasma membrane proteomics and its application in clinical cancer biomarker discovery. *Mole Cell Proteom.* **9**, 1369–1382 (2010). 10.1074/mcp.R900006-MCP200 PMC2938092 20382631

[39] Piotrowski, K. B. et al. ADAM12 expression is upregulated in cancer cells upon radiation and constitutes a prognostic factor in rectal cancer patients following radiotherapy. *Cancer Gene Ther.* **30**, 1369–1381 (2023). 37495855 10.1038/s41417-023-00643-w PMC10581903

[40] Uehara, E. et al. Upregulated expression of ADAM12 is associated with progression of oral squamous cell carcinoma. *Int. J. Oncol.* **40**, 1414–1422 (2012). 22267082 10.3892/ijo.2012.1339

[41] Qiu, L. L. et al. Clinical significance of the interleukin 24 mRNA level in head and neck squamous cell carcinoma and its subgroups: an in silico investigation. *J. Oncol.* **2020**, 7042025 (2020). 10.1155/2020/7042025 PMC7519990 33014054

[42] Laljee, R. P. et al. Interferon stimulated gene - ISG15 is a potential diagnostic biomarker in oral squamous cell carcinomas. *Asian Pac. J. Cancer Prev.* **14**, 1147–1150 (2013). 23621203 10.7314/apjcp.2013.14.2.1147

[43] Gkouveris, I., Nikitakis, N., Aseervatham, J., Rao, N. & Ogbureke, K. Matrix metalloproteinases in head and neck cancer: current perspectives. *Met. Med.* **4**, 47–61 (2017).

[44] Deraz, E. M. et al. MMP-10/stromelysin-2 promotes invasion of head and neck cancer. *PLoS One* **6**, e25438 (2011). 10.1371/journal.pone.0025438 PMC3187776 21998657

[45] Narayan Biswal, B., Das, N., Kumar Das, S., Rath, R. & B. & Alteration of cellular metabolism in cancer cells and its therapeutic. *J. Oral Maxillofac. Pathol.* **21**, 244–251 (2017). 28932034 10.4103/jomfp.JOMFP_60_17 PMC5596675

[46] Mäkinen, L. K. et al. Prognostic significance of matrix metalloproteinase-2, -8, -9, and – 13 in oral tongue cancer. *J. Oral Pathol. Med.* **41**, 394–399 (2012). 22084953 10.1111/j.1600-0714.2011.01110.x

[47] Stott-Miller, M. et al. Tumor and salivary matrix metalloproteinase levels are strong diagnostic markers of oral squamous cell carcinoma. *Cancer Epidemiol. Biomarkers Prev.* **20**, 2628–2636 (2011). 21960692 10.1158/1055-9965.EPI-11-0503 PMC3237810

