# Supplementary material for: Establishing molecular biomarkers for efficient demarcation of tumor and non-tumor tissue in oral squamous cell carcinoma
Source: Sci Rep. 2025 Dec 26;16:3703. doi: 10.1038/s41598-025-33758-1 (PMC12852831; doi:10.1038/s41598-025-33758-1)
Supplement: Supplementary file 1 — Supplementary Material 1 [file 41598_2025_33758_MOESM1_ESM.pdf]

Title: Establishing molecular biomarkers for efficient demarcation of tumor and non-tumor tissue in Oral Squamous Cell Carcinoma

Author's names:

Kashish Gupta[1], Ishan Raval[1*], Apurvasinh Puvar[1*], Shayma Shaikh[1], Madhvi Joshi[1*], Chaitanya Joshi[1*], Siddharth Shah[2], Anand Shah[3], Shashank Pandya[3]

Author's affiliations:

[1] *Gujarat Biotechnology Research Centre (GBRC), Gandhinagar 382011, India*

[2] *Zydus Cancer Hospital, Ahmedabad 380059, India*

[3] *The Gujarat Cancer and Research Institute (GCRI) 380016, Ahmedabad, India*

[*]Corresponding authors:

Dr. Apurvasinh Puvar (Scientist B), Dr. Ishan Raval (Scientist B), Prof. Chaitanya Joshi (Director) and Dr. Madhvi Joshi (Joint Director)

Gujarat Biotechnology Research Centre (GBRC),

DST, GoG, India

Email: apurvasinh.scib@gbrc.res.in, ishan.scib@gbrc.res.in, director@gbrc.res.in, madhvimicrobio@gmail.com

Supplementary table. 1 Sample information/ details.

	Tumour samples	Normal samples	Age	Sex	Stage	Consumption type
1	T_3	N_3	44	Male	Stage-4	Pan-masala
2	T_4	N_4	40	Male	Stage-1	Tobacco
3	T_5		54	Female	Stage-4	None
4	T_6	N_6	55	Male	Stage-4	Tobacco chewing
5		N_7	-	Male	Stage-1	None
6	T_8	-	34	Male	Stage-1	None
7	T_10	-	60	Male	Stage-3	Tobacco, smoking
8	T_11	N_11	50	Male	Stage-2	Tobacco chewing (2 pk/day)
9	-	N_12	64	Male	Stage-4	Bidi, gutkha
10	T_13	N_13	60	Male	Stage-4	Bidi, gutkha
11	T_15	N_15	55	Male	Stage-2	Tobacco chewing
12	T_17	-	51	Male	Stage-1	Tobacco chewing
13	T_18	N_18	44	Male	Stage-2	Tobacco chewing
14	-	N_19	51	Male	Stage-1	Tobacco chewing
15	T_20	N_20	-	Male	Stage-2	Tobacco chewing
16	T_21	N_21	53	Male	Stage-1	Tobacco chewing
17	T_22	-	-	Male	Stage-2	Tobacco chewing
18	T_23	N_23	38	Male	Stage-2	Tobacco chewing
19	T_32	N_32	42	Male	Stage-4	Tobacco chewing
20	T_35	-	42	Male	Stage-1	Tobacco Chewing
21	-	N_37	46	Male	Stage-4	-
22	-	N_38	36	Male	Stage-2	-
23	T_39	N_39	56	Male	Stage-2	-
24	T_40	N_40	37	Male	Stage-4	-
25	T_41	-	53	Male	Stage-1	-
26	T_43	N_43	39	Male	stage-3	-
27	T_45	N_45	49	Male	stage-2	-
28	T_46	N_46	49	Male	stage-1	-
29	-	N_48	46	Male	stage-2	-
30	T_49	N_49	41	Male	stage-2	-
31	T_57	-	71	Male	stage-1	-
32	T_58	N_58	48	Male	stage-4	-
33	T_61	-	40	Male	stage-3	-
34	T_65	N_65	52	Male	-	-
35	T_66	-	-	-	-	-
36	T_67	N_67	-	-	-	-

Supplementary fig. 1 (a) PCA plot of 32 tumor and 27 adjacent normal samples identifying 3 outliers in the dataset. **(b)** PCA plot after removing the identified outliers.

(a)

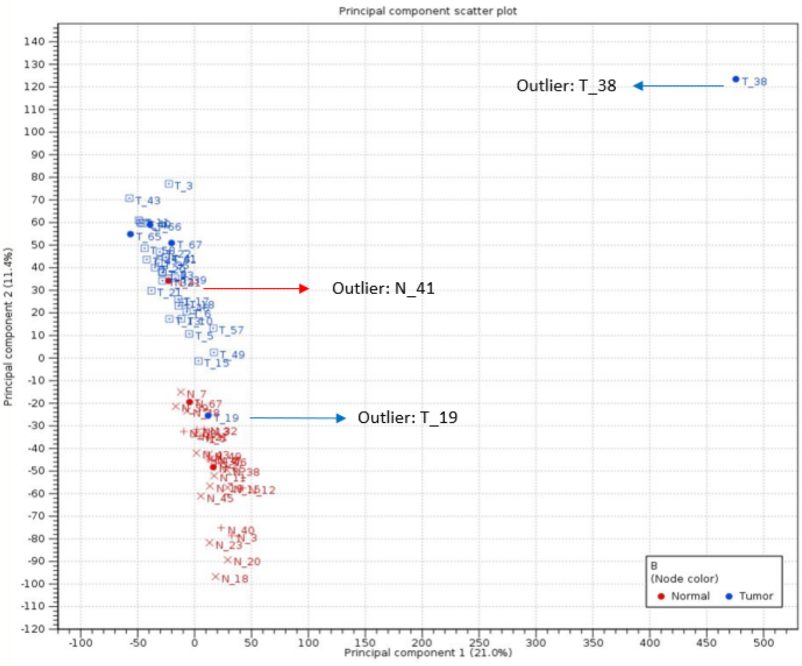

Outliers: T_19, T_38, N_41

(b)

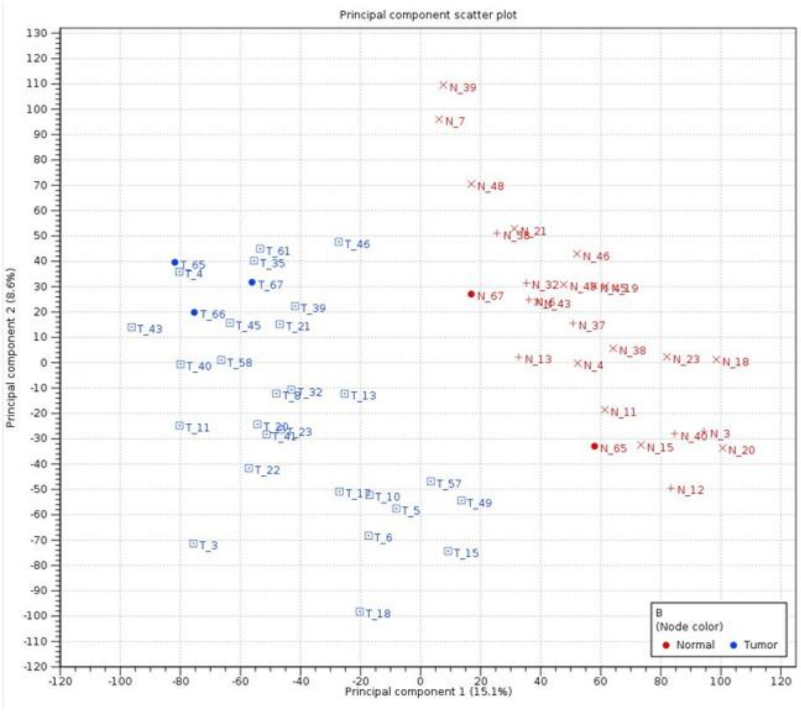

After removing outliers

Supplementary fig. 2 Heat map for visualizing gene expression levels in all the tumor and adjacent normal samples.

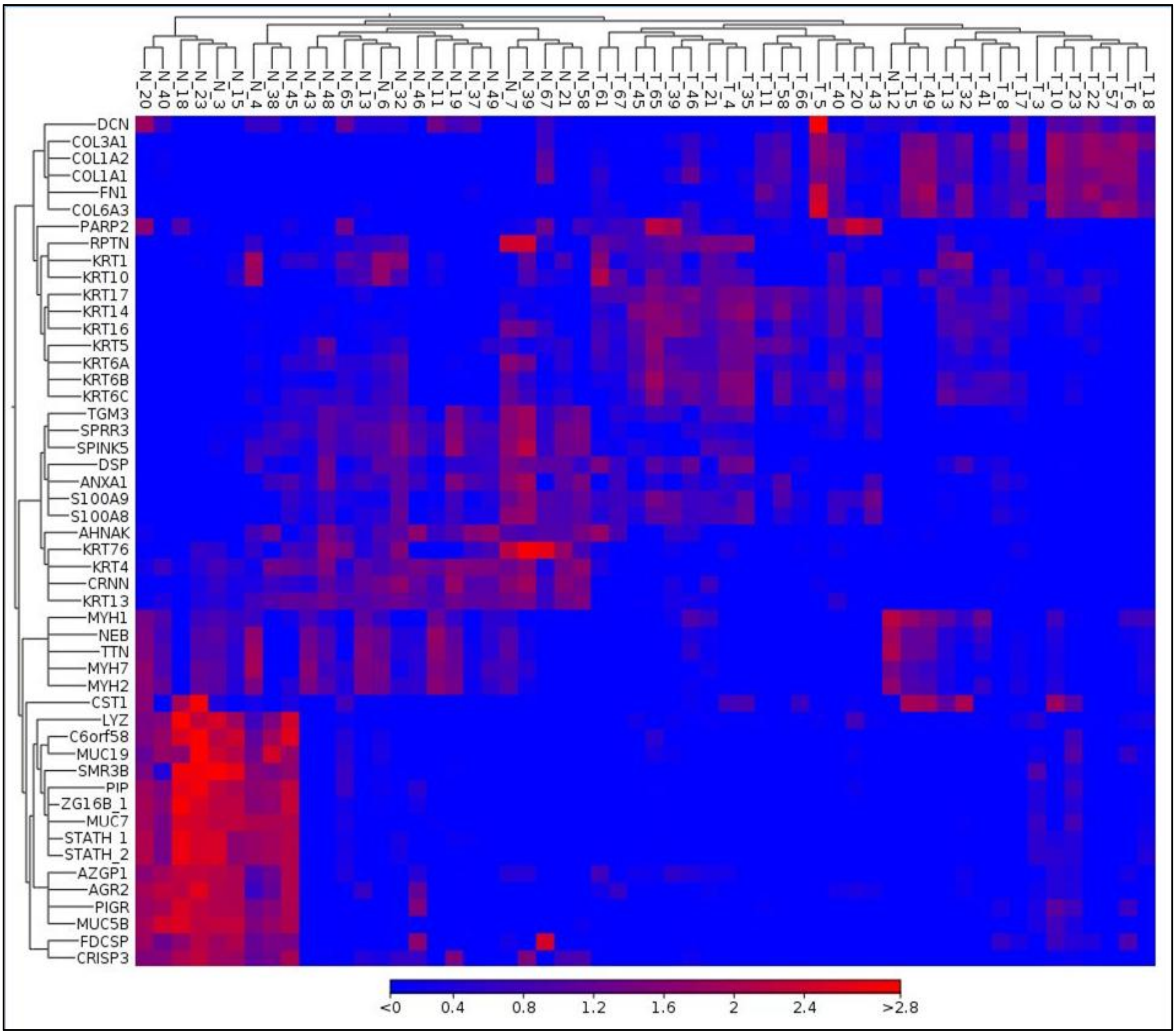

Supplementary table. 2 (a) top 100 upregulated genes and **(b)** top 100 downregulated genes sorted based on their log2 fold change. Genes marked with * are significant in HNSC (based on TCGA database validation).

(a)

	Name	Max group mean	Log2 fold change	FDR p-value
1	*MAGEA6*	5.084	8.683	0.000
2	*MAGEA1*	2.094	8.032	0.000
3	*MAGEC1*	0.425	7.921	0.000
4	*MMP1**	520.547	7.904	0.000
5	*CT45A10*	2.542	7.839	0.000
6	*PTPRN*	1.003	7.504	0.000
7	*TMPRSS15*	0.331	7.498	0.000
8	*GAGE1*	0.608	7.374	0.000
9	*CT45A1*	2.120	7.371	0.000
10	*MAGEA4*	4.724	7.330	0.000
11	*MMP10**	64.527	7.254	0.000
12	*MAGEA3*	4.201	7.249	0.000
13	*CT45A3*	1.386	7.132	0.000
14	*PAGE5*	1.975	6.980	0.000
15	*GAGE12J*	1.537	6.975	0.000
16	*SSX2B*	0.532	6.795	0.000
17	*IL24**	18.206	6.694	0.000
18	*MAGEA9*	0.953	6.693	0.000
19	*MMP13**	74.945	6.676	0.000
20	*MAGEA11*	1.140	6.462	0.000
21	*LHX1_2*	0.217	6.439	0.000
22	*XAGE1A*	1.014	6.373	0.000
23	*SPANXA1*	1.812	6.240	0.000
24	*DEFB4B_1*	23.109	6.235	0.000
25	*HSPA1A_1*	0.300	6.234	0.000
26	*IL11**	3.953	6.163	0.000
27	*GAGE2A*	1.197	6.107	0.000
28	*CDSN_5*	0.743	5.917	0.000
29	*CDSN_4*	0.737	5.901	0.000
30	*SPANXD*	0.863	5.895	0.000
31	*MMP11_1*	13.560	5.871	0.000
32	*SAGE1*	0.157	5.870	0.000
33	*PRAME_1*	0.483	5.829	0.000
34	*MAGEA10*	0.180	5.774	0.000
35	*CSAG3**	2.353	5.758	0.000
36	*CDSN_1*	1.614	5.739	0.000
37	*TDO2_2*	10.161	5.728	0.000
38	*PRAME_2*	0.419	5.678	0.000
39	*HTR2C*	0.115	5.670	0.000
40	*TRIML2*	0.456	5.659	0.000
41	*TDO2_1*	9.756	5.652	0.000
42	*AMTN*	35.367	5.651	0.000
43	*MMP11_2*	13.367	5.620	0.000
44	*IGF2BP1*	0.328	5.603	0.000

45	*MAGEA2*	0.151	5.590	0.000
46	*MMP3**	181.501	5.567	0.000
47	*MAGEA9B*	0.301	5.566	0.000
48	*ISG15**	259.294	5.548	0.000
49	*GAGE12B*	0.788	5.483	0.000
50	*LHX1_1*	0.102	5.436	0.000
51	*CA9**	2.328	5.385	0.000
52	*CCL11*	6.444	5.351	0.000
53	*LIN28B*	0.056	5.323	0.000
54	*LHX5*	0.127	5.269	0.000
55	*CNGB1*	2.705	5.228	0.000
56	*MMP12**	71.452	5.210	0.000
57	*PAEP*	1.064	5.208	0.000
58	*CT45A2*	0.234	5.188	0.000
59	*LOC102723971*	0.152	5.171	0.000
60	*MAGEB2*	1.393	5.146	0.000
61	*CSAG1*	0.384	5.141	0.000
62	*XAGE1B*	0.400	5.066	0.000
63	*GPR50*	0.379	5.047	0.000
64	*DNAH17**	2.084	5.043	0.000
65	*POTEF*	0.192	5.020	0.000
66	*ZP4*	0.143	4.991	0.000
67	*CTAG2*	0.290	4.904	0.000
68	*ADAM12**	8.897	4.900	0.000
69	*MAGEA2B*	0.096	4.900	0.000
70	*GAGE13*	0.496	4.881	0.000
71	*CSF2*	6.511	4.860	0.000
72	*FGG*	0.084	4.760	0.000
73	*CXCL6*	15.278	4.758	0.000
74	*CT45A8*	0.217	4.738	0.000
75	*METTL11B*	0.099	4.738	0.000
76	*GAST*	1.409	4.667	0.000
77	*SPANXB1*	0.944	4.638	0.000
78	*SLC37A4_1*	0.073	4.638	0.000
79	*DAZ3*	0.066	4.584	0.000
80	*KLF17*	0.057	4.569	0.000
81	*CXCL11**	37.445	4.561	0.000
82	*CDSN_6*	7.655	4.557	0.000
83	*NETO1*	0.232	4.536	0.000
84	*HOXC11*	0.248	4.512	0.000
85	*LOC105375816*	0.109	4.494	0.000
86	*CER1*	0.185	4.467	0.000
87	*S100A7A**	23.728	4.455	0.000
88	*HOXA9*	0.187	4.439	0.000
89	*KHDC1L**	3.978	4.426	0.000
90	*GRP*	0.151	4.420	0.000
91	*KRT82*	0.062	4.407	0.000
92	*SOST*	1.449	4.373	0.000
93	*PI15*	11.585	4.356	0.000
94	*BEST2*	0.242	4.353	0.000
95	*BRDT*	0.045	4.340	0.000
96	*COL22A1*	1.448	4.337	0.000
97	*DAZ4*	0.042	4.327	0.000

98	*MLXIP_2*	0.019	4.275	0.000
99	*SOHLH1*	0.032	4.270	0.000
100	*NLRP7_3*	0.081	4.259	0.000

(b)

	Name	Max group mean	Log2 fold change	FDR p-value
1	*PRR27_1*	10.980	-10.709	0.000
2	*ZG16B_1*	2841.185	-10.598	0.000
3	*ZG16B_2*	2845.669	-10.578	0.000
4	*PIP**	1426.734	-10.546	0.000
5	*PRR4_2*	467.942	-10.339	0.000
6	*LPO*	29.192	-10.315	0.000
7	*C6orf58*	176.418	-10.252	0.000
8	*SMR3B*	678.331	-10.170	0.000
9	*STATH_1*	1133.550	-10.100	0.000
10	*PRR4_3*	469.622	-10.088	0.000
11	*SLC13A2*	2.559	-10.064	0.000
12	*PRR27_2*	11.302	-9.983	0.000
13	*CLDN22*	3.324	-9.936	0.000
14	*PRR4_1*	467.381	-9.934	0.000
15	*KRT36*	41.508	-9.836	0.000
16	*STATH_2*	1127.723	-9.733	0.000
17	*OPRPN*	79.627	-9.661	0.000
18	*MUC5B*	162.193	-9.284	0.000
19	*MUC19*	14.255	-9.208	0.000
20	*LACRT*	16.915	-9.150	0.000
21	*MUC7*	344.789	-9.130	0.000
22	*ADIPOQ*	7.245	-9.096	0.000
23	*CIDEC*	4.650	-9.066	0.000
24	*HTN1_1*	128.625	-8.995	0.000
25	*PLIN1*	12.508	-8.929	0.000
26	*TFF3**	131.549	-8.626	0.000
27	*CRISP3**	504.455	-8.597	0.000
28	*HTN1_2*	125.931	-8.596	0.000
29	*TFF1*	15.588	-8.537	0.000
30	*KRT76*	116.356	-8.490	0.000
31	*ITLN1*	14.637	-8.462	0.000
32	*MUC21_7*	9.481	-8.433	0.000
33	*CST4*	58.055	-8.340	0.000
34	*SCGB1D2*	5.274	-8.334	0.000
35	*TTR*	12.384	-8.242	0.000
36	*KRTAP13-2*	4.048	-8.231	0.000
37	*BPIFB2*	266.892	-8.184	0.000
38	*SCGB2A2*	23.066	-8.168	0.000
39	*CAPN9*	1.356	-8.111	0.000
40	*GP2*	1.433	-8.107	0.000
41	*HMGCS2*	11.063	-8.071	0.000
42	*LRRC31*	0.806	-8.035	0.000
43	*CST2*	15.410	-8.021	0.000

44	*SCGB3A1**	52.109	-7.991	0.000
45	*LRRC26*	1.957	-7.952	0.000
46	*CST5*	2.189	-7.921	0.000
47	*PPP1R1B**	11.291	-7.888	0.000
48	*PRB3_1*	3.821	-7.870	0.000
49	*DMBT1*	45.352	-7.737	0.000
50	*ADH1B**	18.877	-7.687	0.000
51	*ODAM*	22.801	-7.600	0.000
52	*WIF1*	3.546	-7.597	0.000
53	*KRTAP13-1*	2.412	-7.593	0.000
54	*MGAM2_2*	0.270	-7.573	0.000
55	*MUC21_1*	58.839	-7.573	0.000
56	*TRARG1*	1.233	-7.565	0.000
57	*MYOC*	5.881	-7.468	0.000
58	*TSPAN8*	22.127	-7.420	0.000
59	*PIGR**	543.607	-7.395	0.000
60	*KRT4**	5263.881	-7.326	0.000
61	*HEPACAM*	0.416	-7.309	0.000
62	*BPIFB6*	0.675	-7.138	0.000
63	*CHRM1*	3.444	-7.074	0.000
64	*KRT85*	1.480	-7.072	0.000
65	*MUC21_4*	27.141	-7.068	0.000
66	*SPDEF*	7.887	-7.065	0.000
67	*SCGB1D1*	1.984	-7.049	0.000
68	*CSN3*	1.352	-7.036	0.000
69	*CSN2_1*	0.789	-7.012	0.000
70	*MUC21_2*	27.207	-7.005	0.000
71	*KRTAP3-1_1*	1.451	-6.984	0.000
72	*PRB3_2*	3.887	-6.969	0.000
73	*DCT*	9.186	-6.936	0.000
74	*KRT84*	35.592	-6.868	0.000
75	*KRTAP3-3_1*	1.044	-6.781	0.000
76	*KRTAP3-3_2*	1.035	-6.774	0.000
77	*SCGB2A1*	5.626	-6.748	0.000
78	*LMAN1L*	1.668	-6.747	0.000
79	*TOX3*	0.738	-6.679	0.000
80	*ERN2*	2.817	-6.660	0.000
81	*DHRS7C*	4.676	-6.647	0.000
82	*CLCNKB*	0.557	-6.614	0.000
83	*RBP4*	2.404	-6.604	0.000
84	*MUC22_4*	0.113	-6.594	0.000
85	*KLK1*	10.935	-6.511	0.000
86	*FAM3B**	13.387	-6.505	0.000
87	*TMEM213*	1.193	-6.468	0.000
88	*MUC21_3*	10.096	-6.456	0.000
89	*KRTAP3-2_2*	1.307	-6.450	0.000
90	*GRIA2*	0.596	-6.434	0.000
91	*ENTPD8*	0.855	-6.424	0.000
92	*FLOT1_2*	0.954	-6.419	0.000
93	*NPY5R*	0.119	-6.403	0.000
94	*KRTAP3-2_1*	1.232	-6.375	0.000
95	*ELAPOR1*	17.254	-6.349	0.000
96	*FDCSP*	453.363	-6.339	0.000

97	*AGR2*	212.646	-6.319	0.000
98	*DNASE2B*	0.456	-6.316	0.000
99	*KRTAP3-1_2*	1.502	-6.314	0.000
100	*C14orf180_2*	0.196	-6.155	0.000

Supplementary fig. 3 (a) PPI network and K-mean clustering for the upregulated genes. Out of the 704 upregulated genes, 217 genes are in Cluster 1 (red), 177 genes are in Cluster 2 (green), and 170 genes are in Cluster 3 (blue).
(b) for each of these three different clusters, the top 20 nodes were identified through Cytohubba in Cytoscape. Marked with * are genes significant in HNSC and marked with ** are genes significant in HNSC present in the top 100. Cluster 1 contains a significant number of genes associated with HNSC.

(a)

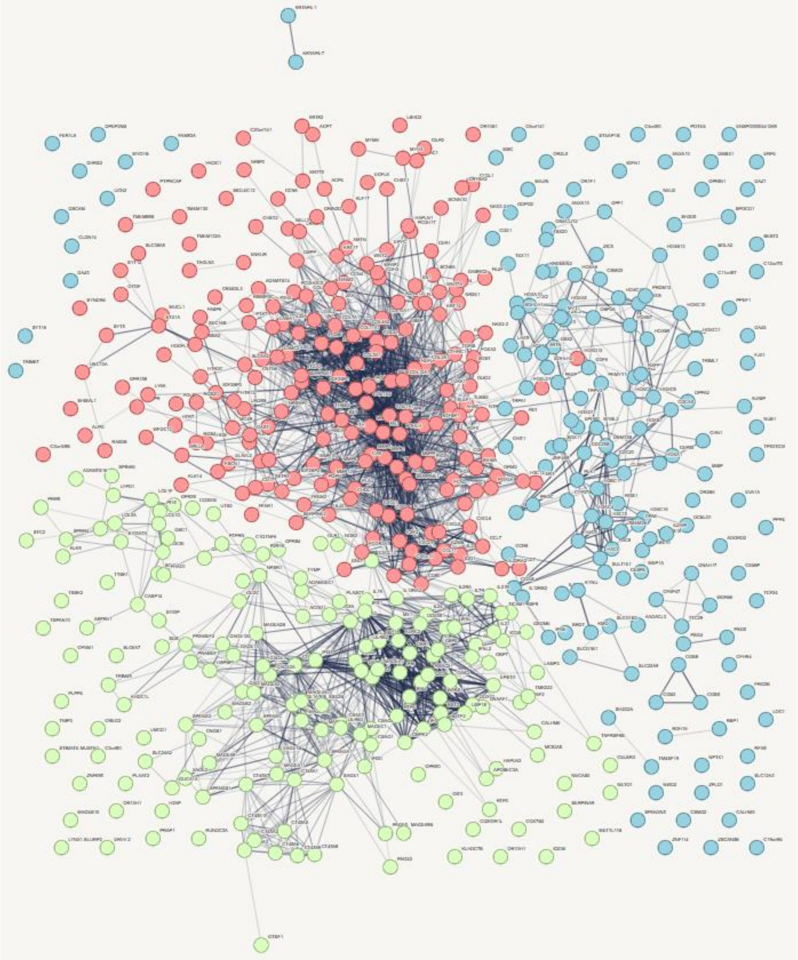

Clusters:		
Color	Cluster ID	Gene count
Red	Cluster 1	217
Green	Cluster 2	177
Blue	Cluster 3	170

(b)

Cluster 1

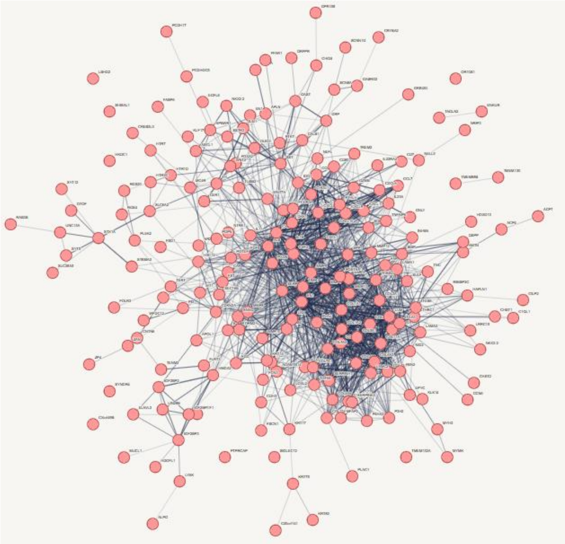

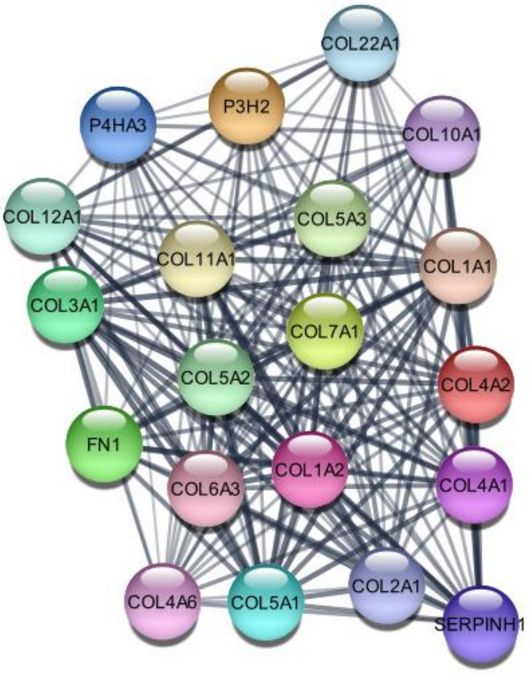

Cluster 1: Cytohubba (top 20 nodes)

P4HA3, COL10A1, COL3A1*, COL5A2*, COL6A3*, COL5A1*, COL11A1*, COL2A1, COL5A3, COL4A2*, COL4A6*, COL22A1, COL4A1*, COL1A2*, COL12A1*, COL7A1*, P3H2*, FN1*, SERPINH1*, COL1A1**

Cluster 2

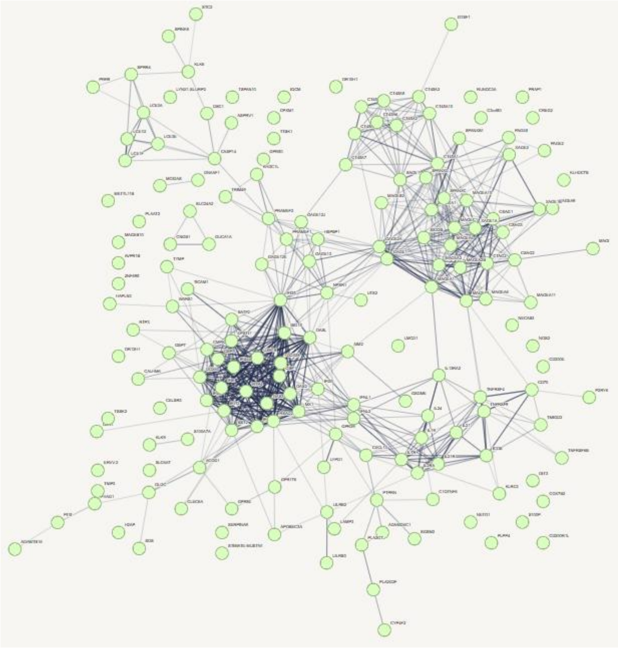

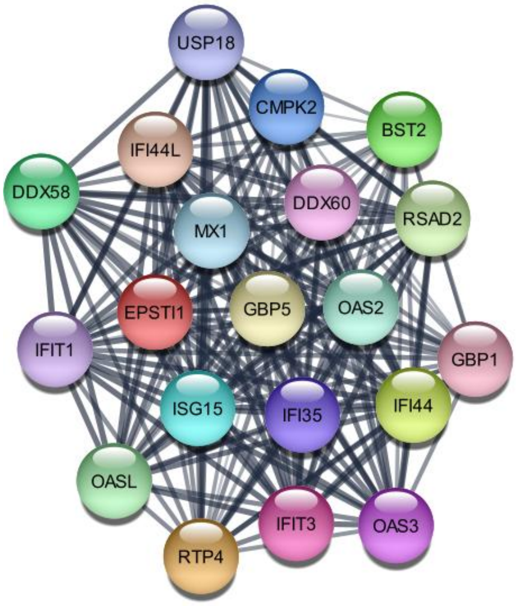

Cluster 2: Cytohubba (top 20 nodes)

GBP5, USP18, MX1, RTP4, IFI35, ISG15**, DDX58, OAS2, EPSTI1*, IFI44*, RSAD2*, GBP1, IFIT3*, BST2*, DDX60, IFI44L, OASL*, IFIT1, OAS3, CMPK2*

Cluster 3

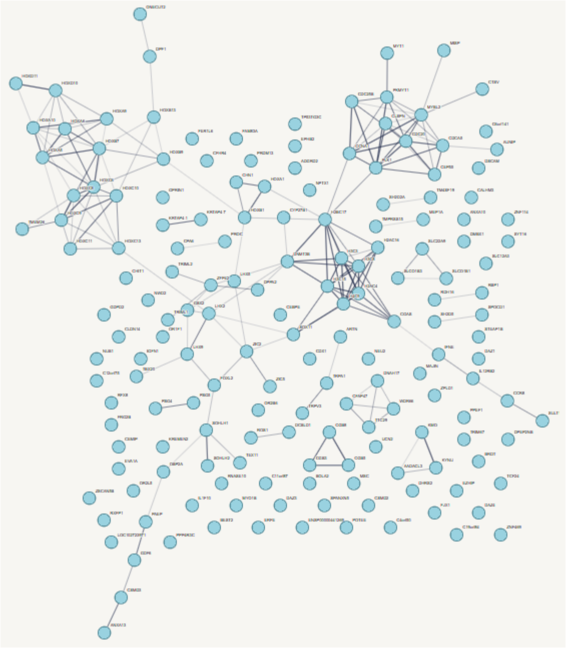

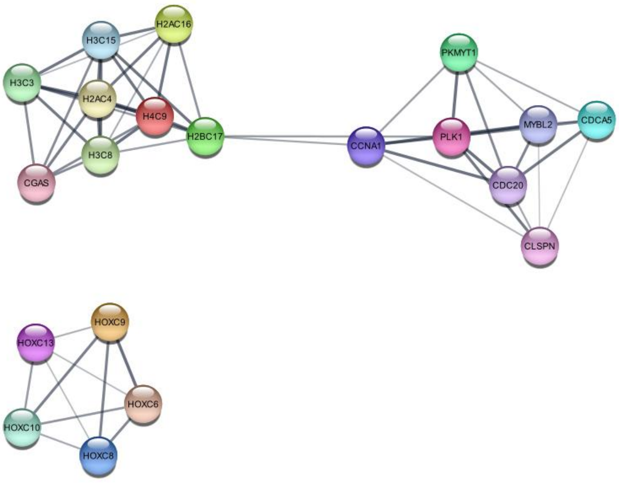

Cluster 3: Cytohubba (top 20 nodes)

HOXC10, HOXC9, H3C8, CDC20, CCNA1, H2AC4, H4C9, HOXC6, H2AC16, HOXC13*, MYBL2*, CLSPN, PKMYT1*, H2BC17, CDCA5*, CGAS, H3C3, PLK1, HOXC8, H3C15*

Supplementary fig. 4 (a) PPI network and K-mean clustering for the downregulated genes. Out of the 1540 downregulated genes, 443 genes are in Cluster 1 (red), 440 genes are in Cluster 2 (green) and 419 genes are in Cluster 3 (blue).
(b) for each of these three different clusters, the top 20 nodes were identified through Cytohubba in Cytoscape. Marked with * are genes significant in HNSC and marked with ** are genes significant in HNSC present in the top 100. Cluster 3 contains a significant number of genes associated with HNSC.

(a)

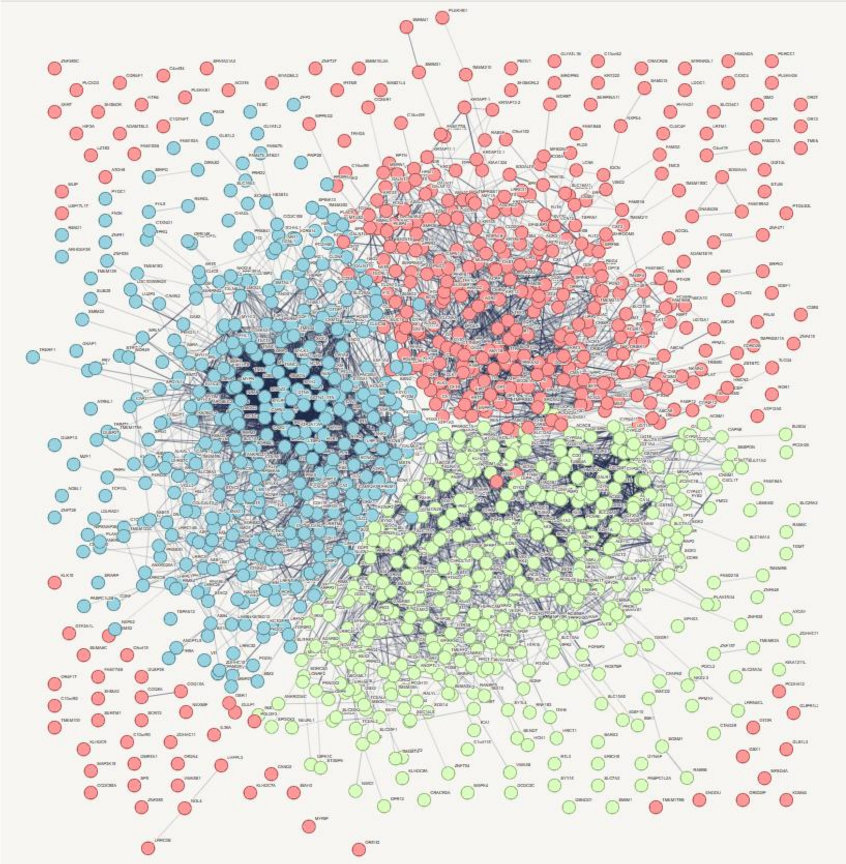

Clusters:		
Color	Cluster ID	Gene count
Red	Cluster 1	443
Green	Cluster 2	440
Blue	Cluster 3	419

(b)

Cluster 1

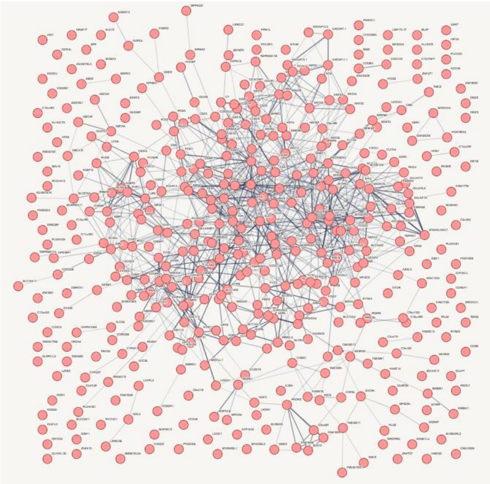

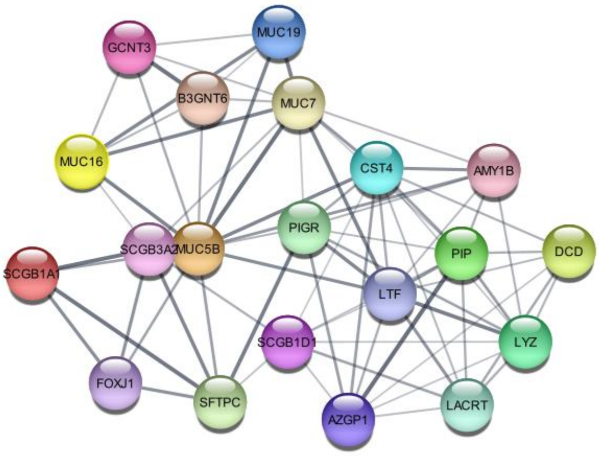

Cluster 1: Cytohubba (top 20 nodes)

AZGP1, GCNT3*, SCGB1A1, MUC16, CST4, MUC5B, LYZ, SFTPC, PIP**, MUC19, MUC7, LTF*, SCGB3A2, LACRT, PIGR**, SCGB1D1, DCD, B3GNT6, AMY1B, FOXJ1*

Cluster 2

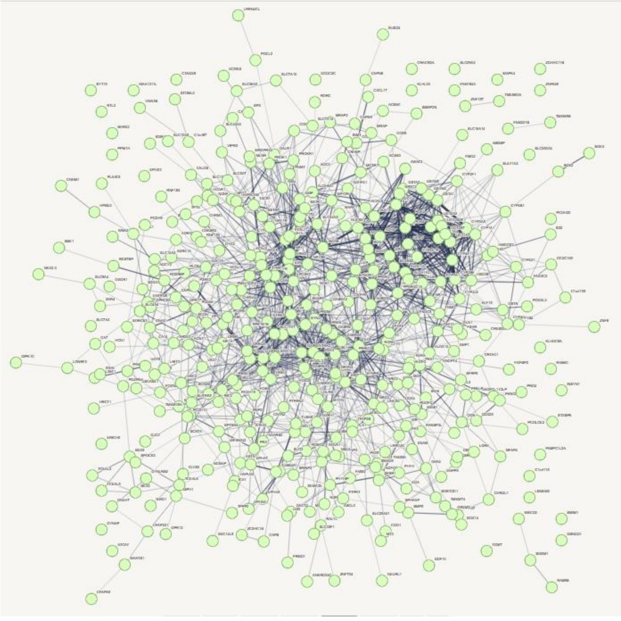

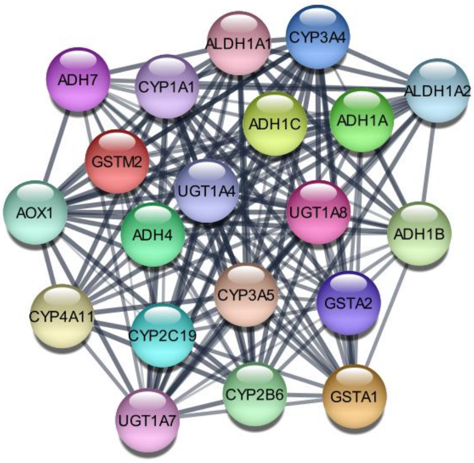

Cluster 2: Cytohubba (top 20 nodes)

*CYP2B6, ADH7**, *ALDH1A1**, *ADH1B***, *GSTM2, UGT1A8, CYP3A4, ADH1A, UGT1A4, ADH4, CYP2C19, CYP1A1, GSTA1**, *ALDH1A2, CYP3A5**, *CYP4A11, ADH1C, GSTA2, UGT1A7**, *AOX1*

Cluster 3

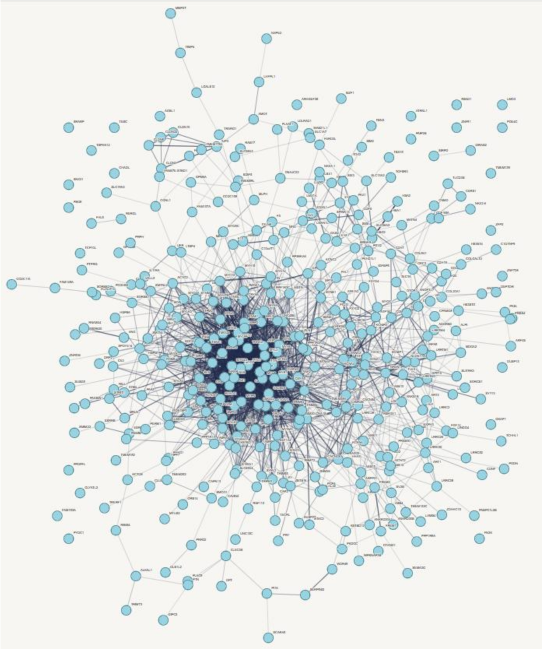

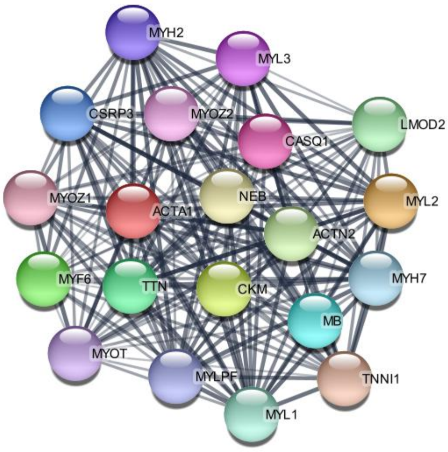

Cluster 3: Cytohubba (top 20 nodes)

MYL3, MYH2, CSRP3*, MYOZ1*, MYF6, CASQ1*, MYOZ2, MB*, MYL2*, MYLPF*, NEB*, MYH7*, TTN, ACTN2*, MYL1*, LMOD2*, TNNI1*, MYOT*, CKM*, ACTA1**

Supplementary fig. 5 Graph showing the number of adjacent normal and tumor samples in each stage of cancer.

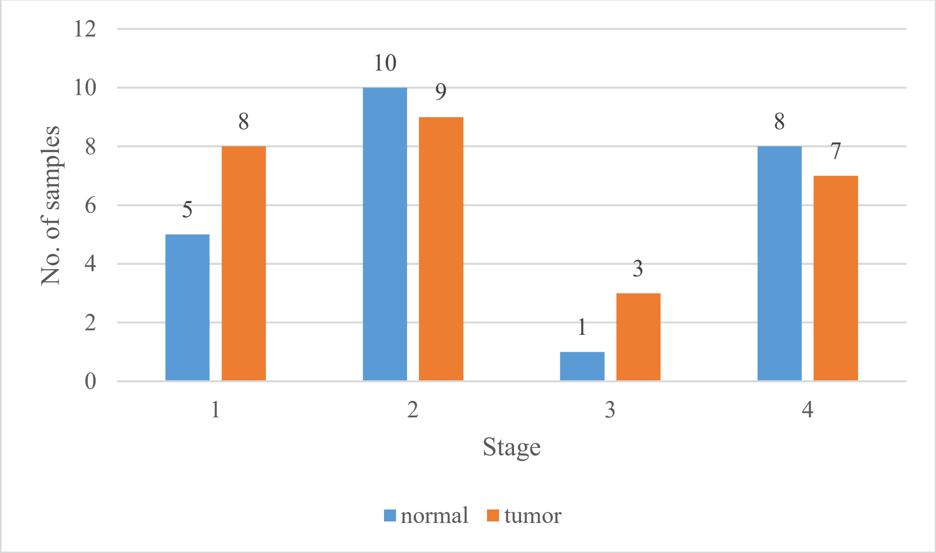

Supplementary table. 3 (a) total number of upregulated and downregulated genes for each of the four stages. **(b)** examining the log2 fold change of the significant upregulated and downregulated genes for each cancer stage, marked with * are the genes with significant log2 fold change i.e. >2 and <-2.

(a)

Stage of cancer	No. of Upregulated genes	No. of downregulated genes
Stage 1	424	967
Stage 2	824	1247
Stage 3	99	194
Stage 4	516	1218

(b)

Upregulated genes				
Gene	Stage 1	Stage 2	Stage 3	Stage 4
MMP1	6.781*	9.612*	10.013*	7.635*
MMP10	5.992*	8.099*	13.213*	7.825*
IL24	5.186*	7.676*	11.700*	6.911*
MMP13	5.701*	9.562*	9.293*	10.387*
IL11	5.939*	6.730*	9.676*	6.150*
CSAG3	3.301*	7.586*	9.583*	6.014*
MMP3	4.541*	6.226*	4.104*	6.210*
ISG15	3.813*	6.395*	6.913*	5.288*
CA9	4.286*	4.524*	5.294*	5.751*
MMP12	3.924*	7.311*	4.529*	5.912*
DNAH17	5.633*	6.060*	6.815*	3.900*
ADAM12	4.758*	5.224*	5.776*	5.601*
CXCL11	3.569*	5.912*	7.233*	4.643*
S100A7A	4.753*	6.957*	3.435*	3.357*
KHDC1L	3.789*	5.581*	8.239*	3.137*

Downregulated genes				
Gene	Stage 1	Stage 2	Stage 3	Stage 4
PIP	-8.754*	-10.743*	-0.680	-8.688*
TFF3	-3.444*	-9.341*	5.277*	-9.270*
CRISP3	-6.453*	-10.289*	3.823*	-8.358*
SCGB3A1	-8.159*	-8.802*	5.842*	-9.075*
PPP1R1B	-6.211*	-9.442*	-1.697	-6.731*
ADH1B	-7.468*	-6.927*	-7.292*	-7.572*
PIGR	-6.765*	-8.995*	6.202*	-6.669*
KRT4	-8.670*	-8.223*	-4.175*	-7.568*
FAM3B	-5.371*	-8.610*	-5.078*	-5.994*

Supplementary table. 4 (a) the number of upregulated and downregulated genes for each of the 20 sample pairs. **(b)** examining the log2 fold change of the significant upregulated and downregulated genes for each sample pair, marked with * are the genes with significant log2 fold change i.e. >2 and <-2.

(a)

	Sample pair	No. of Upregulated genes	No. of downregulated genes
1	N3 & T3	1123	1329
2	N4 & T4	535	1832
3	N6 & T6	1103	1071
4	N11 & T11	1051	1983
5	N13 & T13	494	904
6	N15 & T15	1101	978
7	N18 & T18	1818	1705
8	N20 & T20	1212	2529
9	N21 & T21	833	748
10	N23 & T23	1639	1630
11	N32 & T32	695	897
12	N39 & T39	1843	1154
13	N40 & T40	1122	1692
14	N43 & T43	909	1378

15	N45 & T45	782	1110
16	N46 & T46	1028	1263
17	N49 & T49	1067	924
18	N58 & T58	589	943
19	N65 & T65	811	1977
20	N67 & T67	954	1534

(b)

Upregulated genes																				
Gene	N3 T3	N4 T4	N6 T6	N11 T11	N13 T13	N15 T15	N18 T18	N20 T20	N21 T21	N23 T23	N32 T32	N39 T39	N40 T40	N43 T43	N45 T45	N46 T46	N49 T49	N58 T58	N65 T65	N67 T67
MMP1	10.19*	1.89	12.17*	8.18*	4.78*	8.15*	12.04*	8.78*	9.59*	11.75*	10.41*	5.25*	7.51*	9.59*	6.05*	7.65*	8.74*	7.67*	7.06*	5.90*
MMP10	6.36*	2.78*	9.26*	9.36*	4.39*	5.65*	9.37*	6.01*	7.76*	14.72*	8.62*	9.94*	5.49*	9.97*	6.73*	4.40*	13.36*	8.59*	11.29*	4.40*
IL24	8.01*	2.08*	7.30*	7.42*	3.85*	3.17*	11.468*	10.35*	10.96*	13.68*	10.64*	9.28*	10.88*	11.33*	4.40*	3.62*	8.69*	11.80*	11.35*	5.28*
MMP13	9.01*	-0.67	11.55*	9.35*	8.97*	8.27*	9.30*	10.17*	5.94*	10.03*	12.05*	3.85*	7.20*	6.71*	13.38*	0.24	10.53*	9.20*	7.94*	-4.72*
IL11	5.57*	4.09*	5.23*	10.97*	6.94*	2.30*	10.43*	7.40*	9.71*	7.96*	7.08*	-	8.99*	8.96*	6.11*	3.87*	3.78*	4.46*	5.76*	3.86*
CSAG3	8.31*	2.34*	9.27*	5.45*	-	6.39*	7.88*	8.27*	6.85*	6.30*	-	8.65*	10.22*	9.81*	8.56*	5.46*	11.70*	4.05*	6.65*	-4.90*
MMP3	2.07*	1.22	7.03*	7.51*	5.62*	1.02	8.96*	4.60*	3.00*	8.37*	7.64*	6.09*	4.81*	4.81*	4.31*	6.34*	8.97*	8.63*	5.02*	3.73*
ISG15	4.58*	1.70	5.64*	6.86*	2.49*	6.04*	3.03*	3.68*	4.86*	2.76*	5.50*	10.10*	6.87*	8.41*	6.30*	3.31*	4.92*	3.11*	4.88*	-1.31
CA9	4.17*	8.99*	5.39*	0.75	1.14	-	-	7.47*	-	9.81*	9.49*	8.06*	10.84*	5.51*	-	1.32	-4.16*	5.01*	7.90*	8.22*
MMP12	9.51*	1.86	5.41*	6.32*	2.99*	4.15*	13.80*	7.79*	12.42*	6.89*	7.78*	11.48*	7.78*	5.29*	5.97*	0.37	7.04*	5.27*	6.86*	1.99
DNAH17	6.40*	5.53*	1.75	5.59*	8.17*	3.40*	6.06*	3.88*	6.52*	7.20*	5.52*	9.35*	6.53*	5.99*	6.28*	2.70*	6.58*	6.24*	9.37*	5.68*
ADAM12	5.41*	1.75	7.05*	4.28*	4.92*	5.17*	5.16*	3.88*	4.04*	5.11*	5.43*	3.93*	5.68*	3.34*	2.27*	4.28*	5.96*	4.99*	1.21	2.06*
CXCL11	8.26*	1.97	3.38*	3.09*	0.53	6.23*	12.01*	5.75*	11.72*	2.10*	5.90*	8.10*	12.81*	8.56*	4.78*	8.13*	5.27*	0.88	3.57*	-9.61*
S100A7A	9.28*	0.97	2.18*	9.92*	3.01*	4.48*	4.35*	8.26*	11.66*	10.93*	2.92*	12.48*	10.77*	0.58	7.21*	8.27*	4.84*	11.60*	5.64*	3.82*
KHDC1L	0.97	2.36*	2.45*	-0.23	3.15*	5.53*	5.22*	7.44*	8.16*	8.58*	-0.09	8.87*	7.63*	8.80*	7.03*	9.36*	6.41*	7.07*	9.56*	6.24*

Downregulated genes																				
Gene	N3 T3	N4 T4	N6 T6	N11 T11	N13 T13	N15 T15	N18 T18	N20 T20	N21 T21	N23 T23	N32 T32	N39 T39	N40 T40	N43 T43	N45 T45	N46 T46	N49 T49	N58 T58	N65 T65	N67 T67
PIP	-9.30*	-15.00*	-1.34	-	-	-15.99*	-14.97*	-8.13*	-3.89*	-10.20*	-0.67	0.65	-9.83*	1.05	-11.97*	-10.06*	-	-	-2.87*	-
TFF3	-8.37*	-4.44*	-6.61*	-7.21*	-6.69*	-8.29*	-15.01*	-12.19*	-0.37	-8.55*	0.75	0.65	-12.43*	-	-13.87*	-3.85*	-0.47	0.08	-2.18*	-6.87*
CRISP3	-10.09*	-7.95*	-2.19*	-3.33*	-5.53*	-12.61*	-11.86*	-9.83*	-6.84*	-9.45*	-5.27*	-10.52*	-12.47*	1.47	-17.59*	-1.85	-4.68*	-6.46*	0.47	2.58*
SCGB3A1	-11.52*	-10.05*	-	-	-	-11.26*	-13.05*	-7.48*	-	-8.75*	-	4.31*	-11.93*	-	-12.19*	-8.35*	-	-3.22*	-	-
PPP1R1B	-11.79*	-9.31*	-0.20	-5.16*	-4.58*	-11.71*	-14.06*	-9.87*	-7.65*	-9.85*	-5.48*	-1.27	-8.52*	-6.24*	-11.74*	-9.10*	-8.08*	-5.39*	-6.53*	-
ADH1B	-11.55*	-12.62*	-12.16*	-11.59*	-5.91*	-2.13*	-10.09*	-11.98*	-7.48*	-11.24*	-10.52*	-4.06*	-12.74*	-12.75*	-12.19*	-13.59*	-5.73*	-9.25*	-10.95*	-0.08
PIGR	-8.47*	-10.11*	5.15*	2.53*	-2.46*	-14.24*	-12.63*	-10.98*	-0.02	-7.09*	0.86	2.37*	-10.04*	0.09	-10.28*	-11.60*	-2.46*	0.21	-1.48	1.85
KRT4	-6.71*	-6.87*	-5.64*	-10.71*	-7.46*	-9.20*	-4.36*	-7.24*	-9.69*	-6.82*	-7.83*	-8.63*	-3.49*	-9.04*	-5.95*	-7.69*	-10.28*	-12.95*	-7.74*	-3.97*
FAM3B	-5.37*	-3.62*	-5.71*	-9.46*	-9.89*	-11.49*	-7.55*	-12.31*	-4.86*	-7.13*	-5.01*	-11.62*	-8.71*	-4.93*	-11.46*	-12.69*	-12.19*	-10.19*	-3.64*	-6.15*
